# Viral and thermal lysis facilitates transmission of antibiotic resistance genes during composting

**DOI:** 10.1128/aem.00695-24

**Published:** 2024-07-30

**Authors:** Chaofan Ai, Peng Cui, Chen Liu, Jiawei Wu, Yuan Xu, Xiaolong Liang, Qiu-e Yang, Xiang Tang, Shungui Zhou, Hanpeng Liao, Ville-Petri Friman

**Affiliations:** 1 Fujian Provincial Key Laboratory of Soil Environmental Health and Regulation, College of Resources and Environment, Fujian Agriculture and Forestry University, Fuzhou, China; 2 Guangdong Provincial Engineering and Technology Research Center for Agricultural Land Pollution Prevention and Control, Zhongkai University of Agriculture and Engineering, Guangzhou, China; 3 Key Laboratory of Pollution Ecology and Environmental Engineering, Institute of Applied Ecology, Chinese Academy of Sciences, Shenyang, Liaoning, China; 4 Department of Microbiology, University of Helsinki, Helsinki, Finland; Centers for Disease Control and Prevention, Atlanta, Georgia, USA

**Keywords:** viral lysis, metagenomics, antibiotic resistance genes, composting, extracellular DNA

## Abstract

While the distribution of extracellular ARGs (eARGs) in the environment has been widely reported, the factors governing their release remain poorly understood. Here, we combined multi-omics and direct experimentation to test whether the release and transmission of eARGs are associated with viral lysis and heat during cow manure composting. Our results reveal that the proportion of eARGs increased 2.7-fold during composting, despite a significant and concomitant reduction in intracellular ARG abundances. This relative increase of eARGs was driven by composting temperature and viral lysis of ARG-carrying bacteria based on metagenome-assembled genome (MAG) analysis. Notably, thermal lysis of mesophilic bacteria carrying ARGs was a key factor in releasing eARGs at the thermophilic phase, while viral lysis played a relatively stronger role during the non-thermal phase of composting. Furthermore, MAG-based tracking of ARGs in combination with direct transformation experiments demonstrated that eARGs released during composting pose a potential transmission risk. Our study provides bioinformatic and experimental evidence of the undiscovered role of temperature and viral lysis in co-driving the spread of ARGs in compost microbiomes via the horizontal transfer of environmentally released DNA.

**IMPORTANCE:**

The spread of antibiotic resistance genes (ARGs) is a critical global health concern. Understanding the factors influencing the release of extracellular ARGs (eARGs) is essential for developing effective strategies. In this study, we investigated the association between viral lysis, heat, and eARG release during composting. Our findings revealed a substantial increase in eARGs despite reduced intracellular ARG abundance. Composting temperature and viral lysis were identified as key drivers, with thermal lysis predominant during the thermophilic phase and viral lysis during non-thermal phases. Moreover, eARGs released during composting posed a transmission risk through horizontal gene transfer. This study highlights the significance of temperature and phage lysis in ARG spread, providing valuable insights for mitigating antibiotic resistance threats.

## INTRODUCTION

The dissemination of antimicrobial resistance represents a pervasive global threat to public health (1). Natural transformation, a process where bacteria incorporate extracellular DNA (eDNA) into their genomes, has been suggested to play an important but underrated role in the dissemination of antibiotic resistance genes (ARGs) (2). Accumulating evidence suggests that eDNA is prevalent in diverse environments, including livestock manure (3), sewage sludge (4), wastewater (5), and sediment (3), highlighting the need to study the dissemination of eDNA-mediated transfer of ARGs (6). Specifically, natural transformation of extracellular ARGs (eARGs) could enable the dissemination of ARGs between different taxa, increasing the risk of horizontal transfer of ARGs by human and livestock pathogens from commensal bacteria (7). As a result, more research on the fate and transmission risk of ARGs via eDNA in complex microbiomes is required.

Viruses, as the most numerous entities on earth, have significant impacts on ecosystem function and biogeochemical cycles by regulating microbiome structure and functioning (8, 9). Upon infection, lytic bacterial viruses (phages for short) immediately enter a productive replication cycle, resulting in host cell death and the subsequent release of eDNA into the surrounding environment. Owing to their ubiquity, virus lysis causes extensive microbial mortality across diverse environments (10), leading to nearly 40% daily mortality of prokaryotes in marine environments (11). In theory, viral lysis could enrich ARGs in the environment in the form of eDNA that could be picked up and incorporated into the genomes of other bacteria even if they cannot be infected by viruses (12). This selection process could be facilitated in the presence of antibiotics as well as other antimicrobials (e.g., herbicides, heavy metals, anti-inflammatories, etc.), resulting in the enrichment of antibiotic-resistant bacteria (ARB) (13). However, the role of viruses in releasing ARGs from lysed bacterial cells and their effect in promoting the spread of ARGs via the transformation of eDNA have received little attention.

Thermophilic composting, a commonly used approach for converting livestock manure into organic fertilizer, has been recommended as an efficient method for reducing ARG prevalence (14). While high temperature is thought to be responsible for killing ARG-carrying pathogens during thermophilic composting (14), its effects on eARG formation and subsequent transmission risks are unclear. In addition to temperature, phages could also be responsible for promoting the release of ARG-containing eDNA, as exemplified by our previous study, where we showed that lytic viruses were active and abundant during all phases of hyperthermophilic composting (15). We therefore hypothesized that thermal and viral lysis could potentially accelerate eARG formation and horizontal transfer of ARGs between bacterial species by directly infecting and killing ARB cells during composting (16). This could also explain, at least in part, why substantial amounts of eARGs are still present in the composting end products (17), potentially explaining the rebound of ARGs during compost storage (18).

To study this, we investigated the roles of viral and thermal lysis in eARG release and dissemination during composting by quantifying the abundance, diversity, and composition of eARGs and intracellular ARGs (iARGs) using ethidium monoazide (EMA) method (19). Metagenomic and viromic analyses were further used to link changes in viral and bacterial communities with eARG formation during composting. Finally, we used direct experimentation to show that eARGs released during composting can be taken up by other bacteria via natural transformation and that eARGs are found in different phylogenetic backgrounds at different phases of composting. Our results highlight the importance of both temperature and viruses for eARG release during composting, providing new insights into the spread of ARGs in microbiomes through natural transformation.

## MATERIALS AND METHODS

### Pilot-scale experimental setup for cow dung composting

Thermophilic composting experiments were conducted using a pilot-scale composting plant as described in a previous study (20). Briefly, the raw composting materials consisted of 63 kg of fresh dairy cattle manure (85.6% water content), 28 kg of mushroom residue (32.5% water content), and 4 kg of rice husk (37.6% water content). All raw materials were collected from a nearby dairy cattle factory (Fujian Province, China). Based on the oral reports of the dairy workers, no antibiotic-related drugs were used in the dairy and mushroom production processes. After mixing manually, the raw composting mixture was loaded into three independent plastic foam barrels (270 L volume, 1.5 m diameter, and 2 m height) at 1.2 m bulk height (*N* = 3). Forced aeration was supplied from the bottom of the barrels with a flow rate of 1.5 L/kg·min for 1 hour for every 2 hours. The composting experiments lasted for 45 days with three replications. To monitor the composting temperature, three PT100 thermocouples (PT100; Shanghai Chekon Instrument Co., Ltd., China) were placed at a depth of 50 cm to record changes in average temperature.

### Sample collection

Samples were collected on days 1, 2, 4, 14, 26, and 38 after the start of the composting to cover three typical composting phases: initial phase (day 1: 35°C), thermophilic phase (from day 4 to 20: >50°C), and maturation phase (from day 21 to 45: <50°C, Fig. S1). All samples were collected at a depth of 40–50 cm near the PT100 thermocouples. To obtain well-distributed and homogenized samples, five subsamples (500 g each) per replicate segment were collected from the same depth (40–50 cm), homogenized, and divided into two aliquots. One aliquot was stored at 4°C for analyzing the physicochemical parameters, while the other fresh samples were used to extract DNA immediately for genetic analyses. We chose this sampling approach at the same location in the different composting techniques to reduce the error caused by the heterogeneity of original substrates.

### Extraction of extracellular, intracellular, and total DNA during composting

The extraction of eDNA was performed using previously described and optimized methods (21). Briefly, 0.5 mL of saturated phosphate buffer (Na_2_HPO_4_, 0.12 M, pH 8) was mixed with 0.2 g of fresh composting sample and vortexed for 25 min to desorb adsorbed eDNA in organic matters. The mixed samples were centrifuged at 10,000 × *g* for 10 min at 4°C after supernatants were filtered through a 0.22 µm filter (Millipore, USA) to obtain the extracellular fraction for the subsequent eDNA extraction using a DNA isolation kit (MoBioLaboratories, CA, USA). It should be noted that phosphate-buffered saline (PBS) cannot extract all eDNA from a soil-like matrix due to some tightly adsorbed eDNA in organic matters.

A new DNA extraction technique to remove free, extracellular DNA by using light-activated ethidium monoazide was used to extract the intracellular DNA (iDNA) described previously (22, 23). As demonstrated previously (19, 24), the EMA-based method could efficiently discriminate between viable and dead bacterial cells in soil environments. Therefore, we used the EMA-based method to detect the iDNA during the composting, as compost is a soil-like matrix. Briefly, 0.2 g of fresh composting sample was homogenized and resuspended in sterile 1.5 mL PBS (pH 7.4). EMA solution was immediately mixed with the samples at a final concentration of 100 µg/mL in a dark room before vortexing gently for 5 min. After dark room treatment, the EMA-treated samples were placed on ice and exposed to a 650 W halogen lamp for five consecutive 1-min light/1-min dark cycles. The distance between the sample and the light source was maintained at 20 cm during the light treatment to prevent DNA degradation. Light-treated samples were used for iDNA extraction. Prior to the experiment, we conducted many pre-experiments with compost matrix to explore the optimal experimental parameters (including exposure time, EMA concentration, etc.). Although EMA-based approaches are thought to efficiently discriminate between metabolically active and inactive cells, it also have some drawbacks in turbid sample preparations, such as soils. The effectiveness of EMA binding of eDNA may be reduced because of EMA adsorption to soil particles or ineffective photoactivation of EMA. We also ran a parallel blank control experiment in which the EMA reagent was replaced with distilled water and found no significant difference in their DNA extraction efficiency. This suggests that, in this study, the effect of EMA compost DNA extraction was negligible. To enable comparisons of eDNA amounts across samples, we treated all soil samples the same way.

The total DNA (tDNA) extraction was performed for 0.2 g fresh samples by following the manufacturer’s protocol using the Soil DNA Isolation Kit (MoBio Laboratories, Carlsbad, CA, USA; standard protocol). The eDNA, iDNA, and tDNA samples were obtained for three replicates per sequenced sampling time point: initial phase (day 1; In), thermophilic phase (day 4; Th), and maturation phase (day 26; Ma) of composting. The DNA concentrations were measured using a NanoDrop ND-2000 spectrophotometer (Thermo Fisher Scientific, Wilmington, MA, USA), and DNA quality was assessed using 1% agarose gel electrophoresis and stored at −80°C until further analysis (25).

### Quantitative PCR for estimating the relative densities of antibiotic resistance genes and mobile genetic elements during composting

To track the abundance dynamics of intracellular and extracellular ARGs/mobile genetic elements (MGEs) during composting, quantitative PCR (qPCR) was used to quantify the abundance of 27 widespread ARGs and five common MGEs (Table S1) (14, 26). The 16S rRNA gene was also quantified with qPCR to normalize the abundances of ARGs/MGEs with the bacterial cell abundances. The qPCR was carried out on a Light Cycler 96 system (Roche, Mannheim, Germany), and all the qPCR assays (primers, annealing temperatures, reaction conditions, and amplification protocols) (14, 18) used for all the target genes are listed in Table S1.

### Analyzing bacterial communities based on 16S rRNA amplicon sequencing

The changes in bacterial community composition and diversity during composting were determined using high-throughput sequencing of the 16S rRNA gene (27). Three replicate DNA samples (iDNA, eDNA, and tDNA) at days 1, 4, and 26 of composting were selected for DNA sequencing, representing the initial (In), thermophilic (Th), and maturation (Ma) phases of composting. The primers 515F (5′-GTGCCAGCMGCCGCGGTAA-3′) and 907R (5′-CCGTCAATTCMTTTRAGTTT-3′) were used to amplify the V4–V5 region of 16S rRNA gene and subjected to sequencing on an Illumina NovaSeq 6000 PE250 platform (Guangdong Magigene Biotechnology Co. Ltd, Guangzhou, China). The raw 16S rRNA gene sequences were processed using QIIME 2 (version 2019.7) (13) and DADA2 pipelines for the raw data sequence quality checking, denoising, and filtering (28). The screened amplicon sequence variants were classified using the QIIME2 naive Bayes classifier trained on 99% operational taxonomic units from the SILVA rRNA database (29) (version 138). Microbial diversity was estimated using alpha diversity (Shannon and observed richness) and community composition using beta diversity (weighted UniFrac distance) indexes based on the q2-diversity pipeline within QIIME2.

### Metagenomic assembly, binning, and abundance analysis

To investigate the dynamics of extracellular, intracellular, and total ARGs and MGEs during composting, we performed metagenomic sequencing for samples collected at days 1, 4, and 26 of composting representing the initial, thermophilic, and maturation phases of composting (*N* = 3 for each phase). We constructed a library with an insert size of 300 bp using approximately 1 µg of DNA from each composting phase and sequenced the libraries using the Illumina HiSeq X Ten platform (Magigene Biotechnology, Guangdong) with PE150 (2 × 150 paired reads). All DNA sequences were trimmed to remove Illumina adaptors and Trimmomatic (version 0.36, score > 30, minimum length > 50) was used to obtain high-quality reads (30). All clean reads per sample were assembled using SPAdes (version 3.13.1) with k-mer parameters of 21, 29, 39, 59, 79, 99, and 119 (31). Metawrap (32) based on MetaBAT2, MaxBin2, and Concoct with default parameters was used to bin all assembled contigs larger than 2 kb. High-quality genomes were obtained by manual refinement using the bin_refine module in Metawrap (32). CheckM (version 1.0.13) was used to compute the completion and contamination of all the bins (33). Metagenome-assembled genomes (MAGs) only with ≥70% completeness and <10% contamination were retained for downstream analysis (34–36), and all MAGs were subjected to dereplication using dRep (version 2.3.2) (37). Taxonomic classifications of MAGs were conducted using the Genome Taxonomy Database (GTDB, release 03-RS86) with GTDB-Tk toolkit (version 0.3.2) and classify workflow (38). Protein amino acid composition has been linked to the temperature at which microbes have optimal growth (39), and as a result, DNA sequence information can be used to predict optimal growth temperature (OGT) (40). The optimal growth temperature of MAG was predicted using the default parameters of Tome (version 1.1) (41), a machine-learning method constructed based on the training data of 21,498 microorganisms. This tool for OGT prediction has good accuracy, which has been validated experimentally by using 54 different bacterial species. All MAGs were annotated using Prodigal (version 2.6.3) with the parameter “-p meta” to obtain gene predictions (42). In total, 178 non-redundant MAGs were obtained and used to construct a phylogenetic tree. The “classify” mode in GTDB-Tk (version 0.3.2, with default settings) was used to compare 120 bacterial reference genomes, and multiple sequence alignments were performed based on these marker genomes (38). Subsequently, FastTree (version 2.1.10) was used to construct a phylogenetic tree based on 120 conserved bacterial single-copy marker genes with default parameters from the reference genomes and MAG sequences (43). The tree file was uploaded to the Interactive Tree of Life (https://itol.embl.de/) for visualization and annotation. ARGs carried by MAGs were identified and analyzed using the resistance gene identifier software (version 5.2.1) (44) with the CARD (version 3.2.5, https://card.mcmaster.ca/home) database (released 9 December 2022); only strict and perfect hits with identity >70% with known ARGs were retained (45). Potential antibiotic-resistant bacteria (pARB) were defined as MAGs carrying at least one ARG in their genomes. Mesophilic pARB were categorized as pARB with an OGT lower than 50°C, while thermophilic pARB were categorized as pARB with an OGT of 50°C or higher.

The relative abundance of MAGs was quantified using the CoverM pipeline (version 0.61, https://github.com/wwood/CoverM) (46), using the coverage of mapped reads on the “genome” mode. After quality control, reads were first mapped to MAGs using “make” command to create BAM files (--percentage_id 0.95 --percentage_aln 0.75). Filtered bam files were then used to generate coverage profiles across samples (--trim-min 0.10 --trim-max 0.90 --min-read-percent-identity 0.95 --min-read-aligned-percent 0.75 -m mean). The coverage of each MAG in the unit of “rpkm” (reads per kilobase of exon per million reads mapped) was directly used for alpha- and beta-diversity analyses (log-transformed matrices were used for Mantel correlations).

### Resistome and mobilome analysis based on metagenomics

The abundance of all ARGs (termed resistome) in metagenomes was analyzed using clean reads as input files using local ARG-OAP (version 3.0) (47). ARG reads were identified against the SARG database at the cutoﬀ of 10^−7^ E-value, 80% identity, and 80% coverage. The SARG database contains 32 ARG types, 2,842 ARG subtypes, and 13,672 non-redundant reference sequences (47). Resistance “types” represent the class of antibiotics to which ARGs confer resistance, while “subtypes” represent individual kinds of ARG, such as subtype *tetA* of the tetracycline resistance gene. The relative ARG and MGE abundances were finally represented as targeted gene copies per 16S rRNA gene in each sample. The analysis of MGEs was the same as with ARGs, except for using the MGE database instead of the SARG database (48). The contig open reading frames (ORFs) were predicted using Prodigal version 2.6.3 with a “meta” model (42). The ARG- and MGE-like ORFs were identified against the SARG and mobileOG-db (49) databases using DIAMOND (version 0.9.29.130) with an E-value ≤10^−10^. An open reading frame was designated as an ARG- and MGE-like sequence if its best DIAMOND (50) alignment against database sequences had at least 80% similarity with a ≥80% query coverage (51). The co-occurrence of ARG- and MGE-like was deemed positive if they were both located in the same contig. BLAST was used to identify the putative horizontal transfer of ARGs between different MAGs according to previous studies (52). If a highly homologous resistance gene occurred at the same time in a phylogenetically distant MAG (>95% sequence identity), we considered that horizontal gene transfer may have occurred for that gene. Bacterial hosts of ARGs or MGEs were predicted by taxonomically assigning metagenomic-assembled contigs using CAT (version 5.0.3) (53).

### Viral DNA extraction and viromics

The viral particles were extracted and concentrated from the same composting samples that were used for bacterial metagenomics using centrifugation and filtration (54). Briefly, 20 g of samples collected at days 1, 4, and 26 of composting was mixed with 900 mL of PBS and shaken at 4°C for 15 min (120 rpm). The compost wash was centrifuged at 5,000 × *g* for 10 min at 4°C. Afterward, the supernatant was filtered through 0.22 µm membrane filters using the tangential flow filtration (TFF) system to separate phages. The phage filtrates were sequentially concentrated three times using TFF system with 100 kDa membrane (Millipore, USA). The phage concentrates were then treated with 20 U DNase I (37°C, 50 min, TransGen Biotech) to remove naked DNA using a TIANamp Virus DNA/RNA Kit (Tiangen, Beijing, China). Sequencing libraries were prepared using the DNA Library Prep Kit V2 and then metagenomic sequencing was performed on the Illumina Novaseq 6000 platform (Illumina, CA, USA) to obtain 150 bp paired-end reads. The quality control of raw reads was conducted by Trimmomatic (version 0.39, score > 30 and length > 36 bases) (30), and the quality-filtered sequences were individually assembled using Spades with the same parameters as in bacterial metagenomic analysis.

### Virus identification, taxonomic classification, host prediction, and viral abundance analysis

Viral contigs larger than 5 kb were recovered from metagenomics assemblies using two viral identiﬁcation tools (15): VirSorter2 (version 2.2.3) (55) and Deepvirfinder (version 1.0) (56). First, DeepVirFinder version 1.0 was run with a loose cutoff (score 0.7 and *P* < 0.05) for maximal sensitivity in detecting viral sequences (56). Second, VirSorter2 version 2.2.1 was used to identify putative viral sequences with scores ≥ 0.95 using DeepVirFinder-output sequences as input files, and the parameter “dsDNAphage, ssDNA” was used to identify whether the virus is a single-stranded or double-stranded DNA virus (55). To remove non-viral sequences during the VirSorter2 analysis, CheckV (version 0.9.0) was used for quality assessment as described previously (57). The final viral contig data set was manually curated and trimmed to remove potential host regions according to a previous protocol (58). Predicted contigs were considered of viral origin if they met at least one of the following three criteria: (i) contigs contained at least one virus-specific hallmark gene; (ii) contigs had VirSorter2 scores ≥ 0.95; or (iii) the total number of genes annotated as “unknown” (egg-NOG version 5.0.0 database) accounted for ≥80% of the total number of genes on the scaffold. Finally, all potential viral contigs were checked using VIBRANT (version 1.2.1, virome mode) with default settings (59). The identified viral contigs were clustered at 95% average nucleotide identity with at least 85% coverage using CD-HIT (version 4.8.1, parameters: -c 0.95 -aS 0.85 -d 400 -T 20 -M 200000 -n 5) (60), resulting in a total of 2,694 viral OTUs (vOTUs). The longest sequence from each cluster was used as a representative sequence of a given viral group in subsequent analyses. Completeness of viral genomes was estimated using the CheckV (version 0.8.1) pipeline, and CheckV and VIBRANT were further used to predict if viral contigs were temperate based on the presence of prophage integration sites or integrase genes (57). PhaGCN2 was used to classify and identify viral sequences at the family level with the parameter len = 5000 (61).

The potential hosts of vOTUs were predicted using computational methods based on CRISPR spacer similarity. The CRISPR spacers were recovered from bacterial MAG metagenomic contigs with CRT (version 1.2), with default parameters (62). CRISPR spacer similarity in the vOTU and MAGs were identified using BLASTn (“blastn-short” mode preset, 100% nucleotide identity, mismatch ≤ 1, and *e*-value ≤ 10^−5^). The 851 vOTUs could be putatively linked to 48 pARBs based on CRISPR spacer similarity. It should be noted that the CRISPR spacer-based method for determining prophage movement requires further experimental verification, as CRISPR spacer matches may only be a historical trace of infection. The relative abundance of vOTUs in metagenomic data sets was quantified using the CoverM pipeline (46) based on the coverage of mapped reads using the “contig” mode and expressed as rpkm.

### Assessing the transmission of ARG from environmental DNA

To investigate the potential transmission risk of ARGs via eDNA extracted from composting samples, we used the samples collected at different phases of composting for natural transformation experiments in the laboratory. Briefly, *Vibrio vulnificus* was used as an example of a naturally competent human pathogenic strain (63) and a recipient of eARGs. A mixture of *V. vulnificus* (10^8^ CFU/mL) with 20 ng/µL eDNA (50 µL; isolated from different phases of composting) was placed in a static incubation at 30°C for 24 hours, followed by a 2-hour incubation at 37°C in fresh LB-N broth. The bacterial suspension was serially diluted and transferred to LB plates containing 4 mg/L tetracycline antibiotic (64). Tetracycline was selected based on the abundance of underlying ARGs observed during the composting experiment. The transformation efficiency was calculated based on the number of transformants per gram of dry compost. In the control treatment, eDNA was replaced with sterilized water, resulting in no successful transformants observed on tetracycline LB plates.

### Isolation of antibiotic-resistant bacteria during composting

A culture-based method was used to compare the number and composition of antibiotic-resistant bacteria during composting. Isolation and identification of culturable ARBs were performed as previously described (18). Briefly, 10 g of fresh sample was mixed with 90 mL PBS buffer and shaken for 30 min at 37°C. Subsequently, 100 µL of serially diluted samples was plated onto LB agar plates supplemented with one of the various antibiotics, including tetracycline (4 µg/mL), chloramphenicol (16 µg/mL), ampicillin (100 µg/mL), kanamycin (100 µg/mL), amoxicillin (64 µg/mL), streptomycin sulfate (30 µg/mL), and gentamicin (10 µg/mL). The experiments were repeated three times for each antibiotic. Antibiotic concentrations were chosen according to the standards of the Clinical and Laboratory Standards Institute for *Escherichia coli* (https://clsi.org/). After incubation at 37°C for 24 hours, culturable ARBs with different colors, morphologies, and transparencies were obtained from the initial (day 1, 23 ARBs were isolated across all antibiotic plates) and maturation (day 26, 24 ARBs were isolated across all antibiotic plates) composting phase samples. The detailed information on isolates is listed in Table S2. The genomic DNA of all isolated ARBs was extracted using the Bacteria DNA Kit (Tiangen, Beijing, China), and the taxonomic identification of isolated colonies was performed by amplifying the 16S rRNA gene using the primers 27F (5-AGAGTTTGATCCTGGCTCAG-3) and 1492R (5-GGCTACCTTGTTACGACTT-3).

### Statistical analysis

The data were statistically analyzed using R (version 4.1.3) (https://www.r-project.org/). The overall mean abundance differences were analyzed using one-way ANOVA followed by multiple comparisons using Tukey’s HSD test or the Student’s *t*-test. Nonparametric PERMANOVA (Adonis function, 999 permutations) was used to determine the significance of sampling time points on the microbiome composition. In non-parametric Wilcoxon signed-rank and Adonis tests, statistical significance was determined based on 999 permutations. The alpha- and (richness, Shannon’s index) beta-diversity (Bray-Curtis distance) analyses associated with microbiomes, resistome, and mobilome were conducted using vegan and ggplot2 packages in R. The vegan package was also used to perform variation partitioning analysis (VPA) based on partial redundancy analysis to assess the relative contribution of composting temperature, viral lysis, and bacterial community for the increase in the proportion of eARGs during composting.

## RESULTS AND DISCUSSION

### Composting is effective in removing ARGs but enriches the proportion of eARGs relative to iARGs

**Fig 1:**
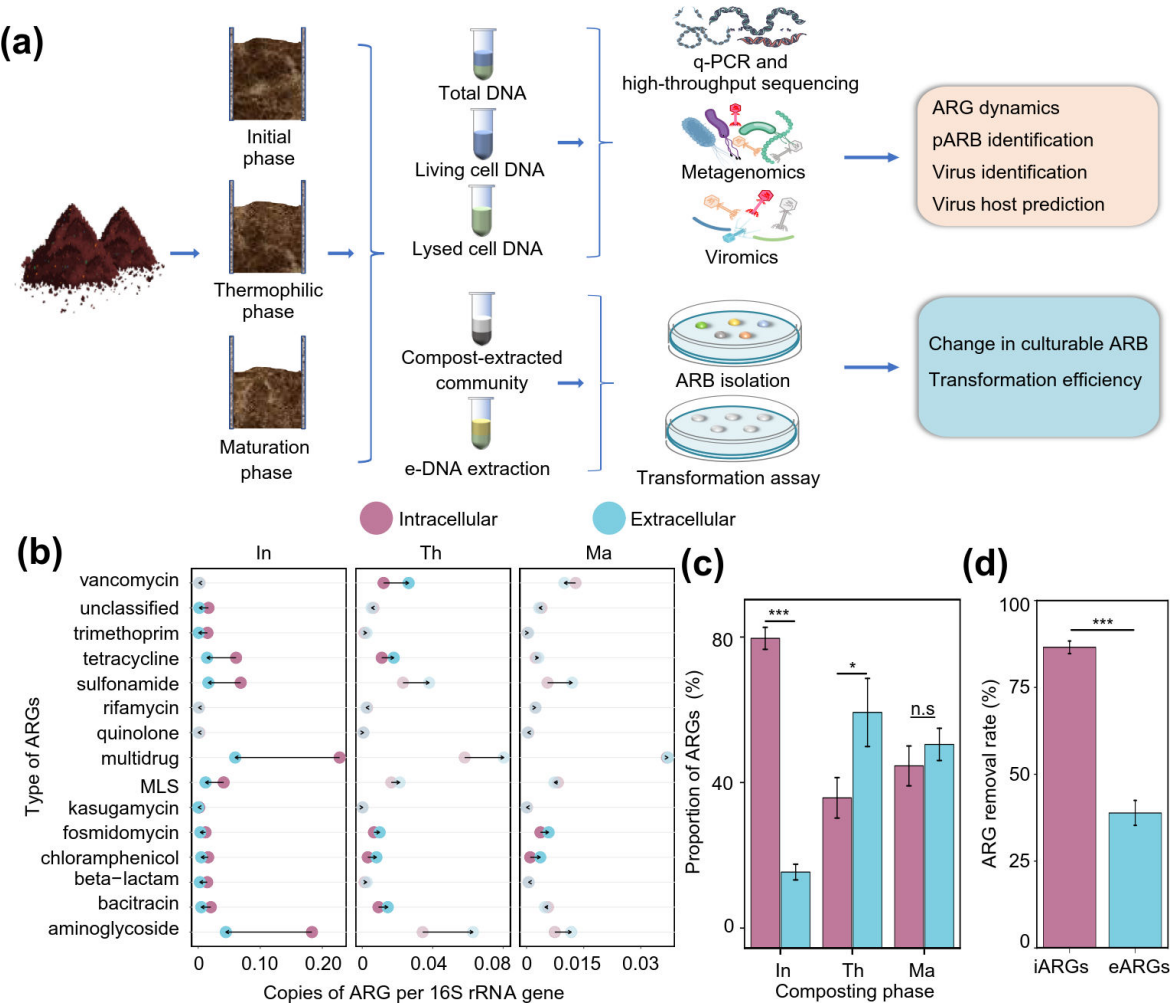
Schematic diagram of the experimental design and variation in the intracellular and extracellular ARGs during composting. (a) Schematic diagram of the experimental design and analysis methods. (b) Dumbbell plots illustrate the abundance differences of intracellular and extracellular ARGs during composting. Dumbbells with dark colors indicate significant differences between groups (*P* < 0.05), while lighter colors indicate non-significant differences (*P* > 0.05). (c) Changes in the proportion of intracellular and extracellular ARGs at different phases of composting. (d) The removal rate of intracellular and extracellular ARGs during composting. All data sets are based on three biological replicates, and significance levels are indicated by * (*P <* 0.05), ** (*P <* 0.01), and *** (*P <* 0.001), while “n.s” denotes non-significant difference (*P* > 0.05). The abbreviations “In,” “Th” and “Ma” denote initial, thermophilic, and maturation phases of composting, respectively.

To investigate the changes in intracellular and extracellular ARGs during composting, we extracted DNA, including tDNA, iDNA, and eDNA, from three phases of composting for qPCR and metagenomics analysis (Fig. 1a). We first used metagenomics to track the dynamics of intracellular and extracellular ARGs during composting. Similar to previous studies (4, 7), composting could effectively reduce the total abundances of ARGs and MGEs based on metagenomics (85.9% and 94.3%, respectively, Fig. S2), possibly due to the degradation of thermolabile pARB by heat. However, we found that large numbers of eARGs and eMGEs were present in composting. In total, 19 ARG types and 376 ARG subtypes (including 3,381 non-redundant ARG genes) were detected, which were associated to multidrug (31.7%), aminoglycoside (21.9%), sulfonamide (11.8%), macrolide-lincosamide-streptogramin (7.4%), tetracycline (6.6%), and vancomycin (5.7%) resistances (Fig. 1b). We found that the proportion of eARGs at different phases of composting showed a clear dynamic response along with the composting phase. For example, the proportion of eARGs was less than 20% in the initial phase samples, while it increased significantly to 61.1% by the thermophilic phase and remained at 52.8% at the maturing phase of composting (Fig. 1c and d). The trend of eMGE dynamics during composting was similar to that of eARG dynamics (Fig. S3), suggesting that ARG and MGE abundances were positively correlated. In contrast, the proportion of iARGs decreased from 80.3% at the initial phase to 47.2% at the end of composting, indicating that the accumulation of eARGs was mainly due to the release of iARGs into the environment. The metagenomic results based on relative abundances were corroborated by qPCR analysis based on absolute abundances (Fig. S4). These results may be attributed to high bacterial mortality and community succession due to high composting temperatures (26). For example, thermophilic composting could reduce the abundance of ARGs by killing the ARG-carrying bacteria. This could, however, lead to the release of eARGs, which pose a serious health risk due to their high transmission stability through natural transformation, potentially leading to widespread ARG dissemination (12). In support of this, we observed significant positive correlations between composting temperature and abundance of eARGs (*R*^2^ = 0.6561*, P* < 0.05, Fig. S5). Taken together, we found that composting effectively removed iARGs but enriched the proportion of eARGs and eMGEs likely due to bacterial cell death during high composting temperatures.

### Temperature-driven bacterial community succession is associated with eARG release during composting

To link changes in eARG release with microbial community dynamics, we characterized the diversity and composition of bacterial communities using high-throughput amplicon sequencing based on iDNA, which considers microbial cells only. Our analysis identified Proteobacteria, Bacteroidota, Firmicutes, Chloroflexi, and Actinobacteriota as the most dominant phyla living in the initial compost material, accounting for 86.9% of all the sequence data (Fig. S6). While these phyla persisted throughout the composting, both bacterial Shannon index (*F*_2,6_ = 34.5, *P* < 0.05, Fig. S7a) and composition (*R*^2^ = 0.7726, *P* < 0.05, PERMANOVA, Fig. S7b) varied significantly between different composting phases. Procrustes analysis revealed significant changes in correlation between bacterial community and eARG (*M*^2^ = 0.3518, *P* < 0.05) and eMGE (*M*^2^ = 0.4468, *P* < 0.05) compositions during the composting (Fig. S8). In addition, we found that the relative abundance of Firmicutes and Verrucomicrobia was significantly correlated with the eARG abundances (Fig. S9). This result indicates that bacterial community succession was associated with eARG release, which is consistent with our previous study where bacterial community composition was linked with the ARG abundances and their dissemination (14).

**Fig 2:**
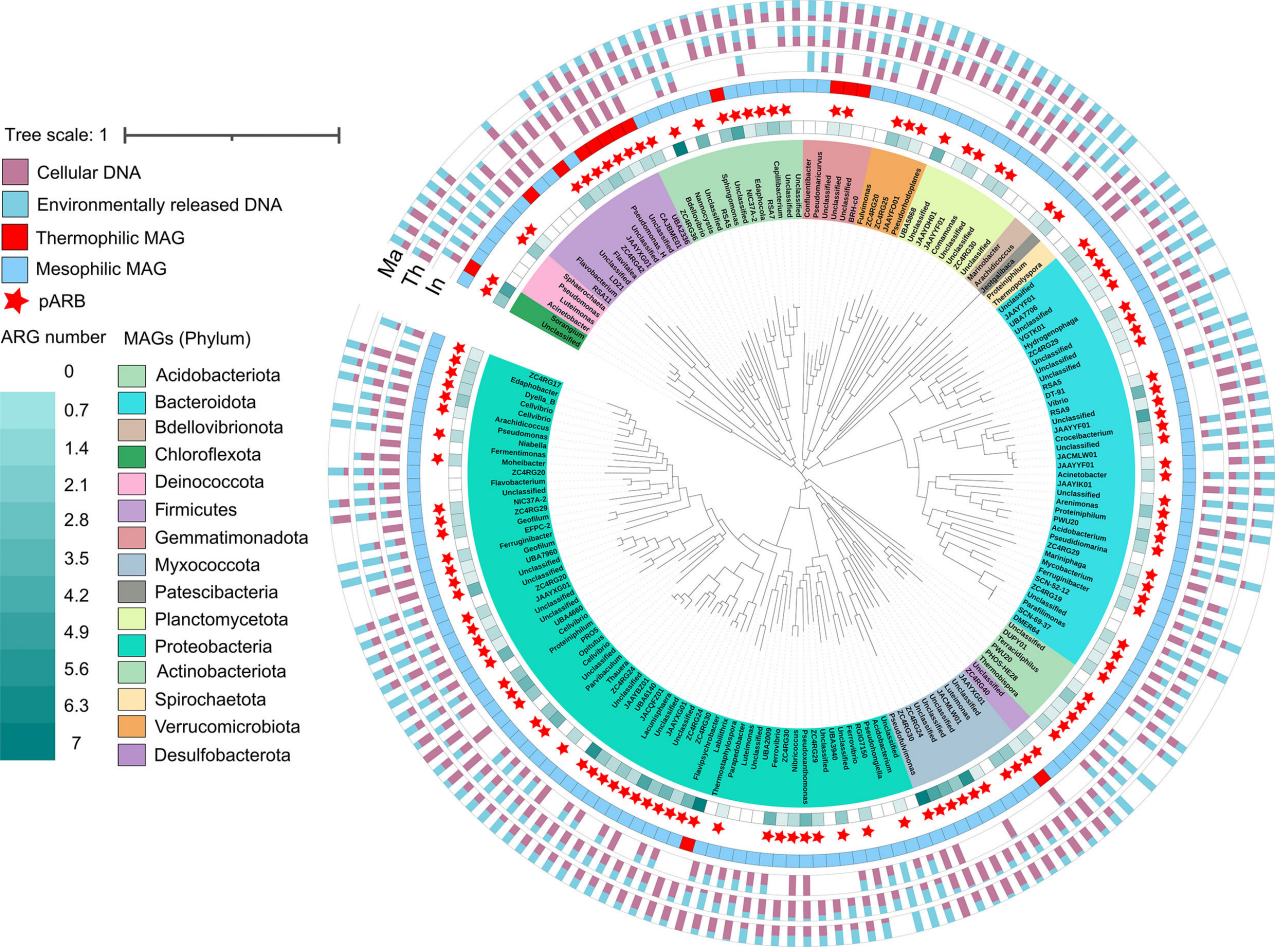
Overview of ARG-carrying bacterial community based on recovered MAGs from metagenomic binning. Phylogenetic tree of 178 non-redundant bacterial MAGs recovered from compost metagenomic samples. The inner circle represents the taxonomic classification of MAGs at the phylum level, while the color shade on the second inner circle indicates the number of ARGs carried by MAGs (legend on the left). The asterisks on the third inner circle indicate the ARG-carrying MAGs, while blue and red colors on the fourth inner circle indicate the optimal growth temperatures of MAGs (mesophilic and thermophilic MAG). The bar graphs shown on the outer circle represent the proportion of cellular and environmental DNA of MAGs at three different phases of composting. The tree scale shows nucleotide substitutions per site. “In,” “Th,” and “Ma” represent the initial, thermophilic, and maturation phases of composting, respectively.

Next, we employed metagenomic binning to identify bacteria associated with the release of eARGs and eMGEs at the MAG level. In total, 178 non-redundant MAGs with completeness >70% and contamination <10% were recovered (65, 66). We used the Tome (version 1.1) to predict the optimal growth temperature of all MAGs based on machine learning method according to a previous study and identified 164 mesophilic (OGT < 50°C, dominated by Proteobacteria, Bacteroidota, and Actinobacteriota) and 14 thermophilic (OGT ≥ 50°C, dominated by Firmicutes and Gemmatimonadota) MAGs (Fig. 2; Table S3). The taxonomic composition of MAGs was consistent with the 16S rRNA data (Fig. S6), indicating that the recovered MAGs reliably represented the composting bacterial community. Of these MAGs, Proteobacteria, Bacteroidota, Actinobacteriota, Acidobacteriota, and Firmicutes were the predominant phyla, comprising 98.3% of all MAGs (Fig. S10). Across all the MAGs, 741 ARGs were detected, and 69.1% (123 of 178) of MAGs were identified as pARB, carrying at least one ARG (Table S4). Importantly, mesophilic pARB had a much higher content of ARGs per MAG compared to thermophilic pARB (5.1 vs 2.8), suggesting that most ARGs were carried by mesophiles. Interestingly, ARGs in mesophilic pARB were more often found in dead bacterial cells (extracellular DNA, Fig. S11), while ARGs in thermophilic pARB were more often found in viable bacterial cell (*P* < 0.05, *t* = 8.5, and df = 4, Fig. S12). A significant relationship between the abundance of mesophilic pARB in the environmental DNA fraction and composting temperature was observed (*R*^2^ = 0.9604*, P* < 0.05, Fig. S13). This is not surprising as mesophilic pARB were likely killed by high composting temperature, leading to an increase in the relative abundance of these mesophilic pARB in the environmental fraction. These findings are in line with a previous study that reported a positive association between eARG abundances and composting temperature during composting (67). As a result, while thermophilic composting is an efficient approach to removing total ARGs from the raw composting materials (14), it seems to release ARGs into the environment as eARGs, which could pose risks for eARG dissemination to surviving bacteria via horizontal gene transfer.

### Viral lysis of pARB facilitates the relative increase of eARGs during composting

**Fig 3:**
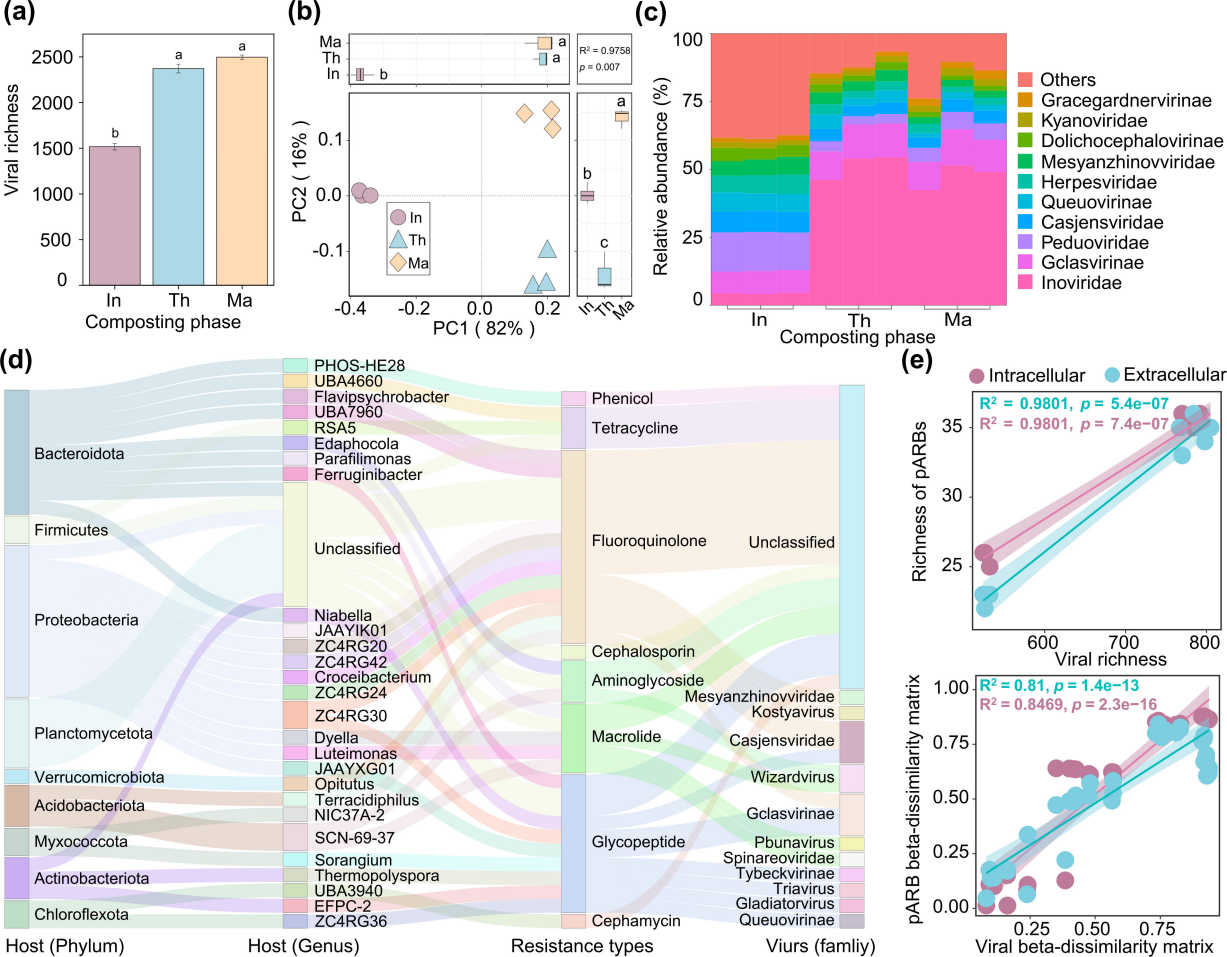
Viral community diversity, composition, abundances, and predicted virus-host interactions during composting. Changes in viral richness (a) and community composition (b and c) during different phases of composting. Analysis in panel b is based on principal component analysis of vOTUs, while analysis in panel c is based on relative vOTU abundances at the family level. (d) Predicted virus-host links between vOTUs and pARB. The left two panels represent pARB host taxonomy colored by phyla and genera, and the right two panels show the types of resistance genes carried by pARB and associated viral families. Gray connecting lines show associations between pARB hosts (at phylum and family level) with types of resistances and their associated viruses. (e) The relationships between pARB and viral community richness (upper panel) and pARB and viral community beta-diversity matrices (lower panel) across all composting samples. In panels a and b, different lowercase letters represent significant differences between groups based on ANOVA test (*P <* 0.05). The shaded area in panel e represents a 95% confidence interval around the fitted regression line. In panels a–c, “In,” “Th,” and “Ma” represent the initial, thermophilic, and maturation phases of composting, respectively.

To investigate whether the formation of eARGs could additionally be explained by the viral lysis of pARB, we characterized the viral community and the associated hosts during composting using the viromic analysis. A total of 2,803 putative viral contigs with sizes greater than 5 kb were obtained from the viral metagenomic assemblies. After clustering and dereplication (95% identification and over 85% coverage), 2,694 vOTUs were retained (Table S5), which were mainly lytic double-stranded DNA viruses (91.5%). Similar to bacteria, we observed an increase in viral richness (*F*_2,6_ = 635.8, *P <* 0.05, Fig. 3a) and change in viral community composition (*P* < 0.05, *R*^2^ = 0.9758, PERMANOVA) during the composting (Fig. 3b). Moreover, the changes in viral community composition were significantly positively correlated with the composting temperature (Fig. S14). The *Inoviridae* (51.5%), *Gclasvirinae* (11.9%), *Peduoviridae* (14.2%), and *Casjensviridae* (7.6%) were the predominant viral families, and all these viruses were bacteriophages (Fig. 3c). Furthermore, the viral and bacterial Shannon indexes (*R*^2^ = 0.7569, *P* < 0.05, Fig. S15a) and community compositions (Bray-Curtis; *R*^2^ = 0.5476, *P* < 0.05, Fig. S15b) were positively correlated. Notably, no viral ARGs were identified in any vOTUs based on the CARD database (44). This suggests that viruses themselves do not carry ARGs, 95% of them are lytic viruses, which is in line with previous studies suggesting that lytic viruses generally carry very few ARGs as accessory genes (68).

**Fig 4:**
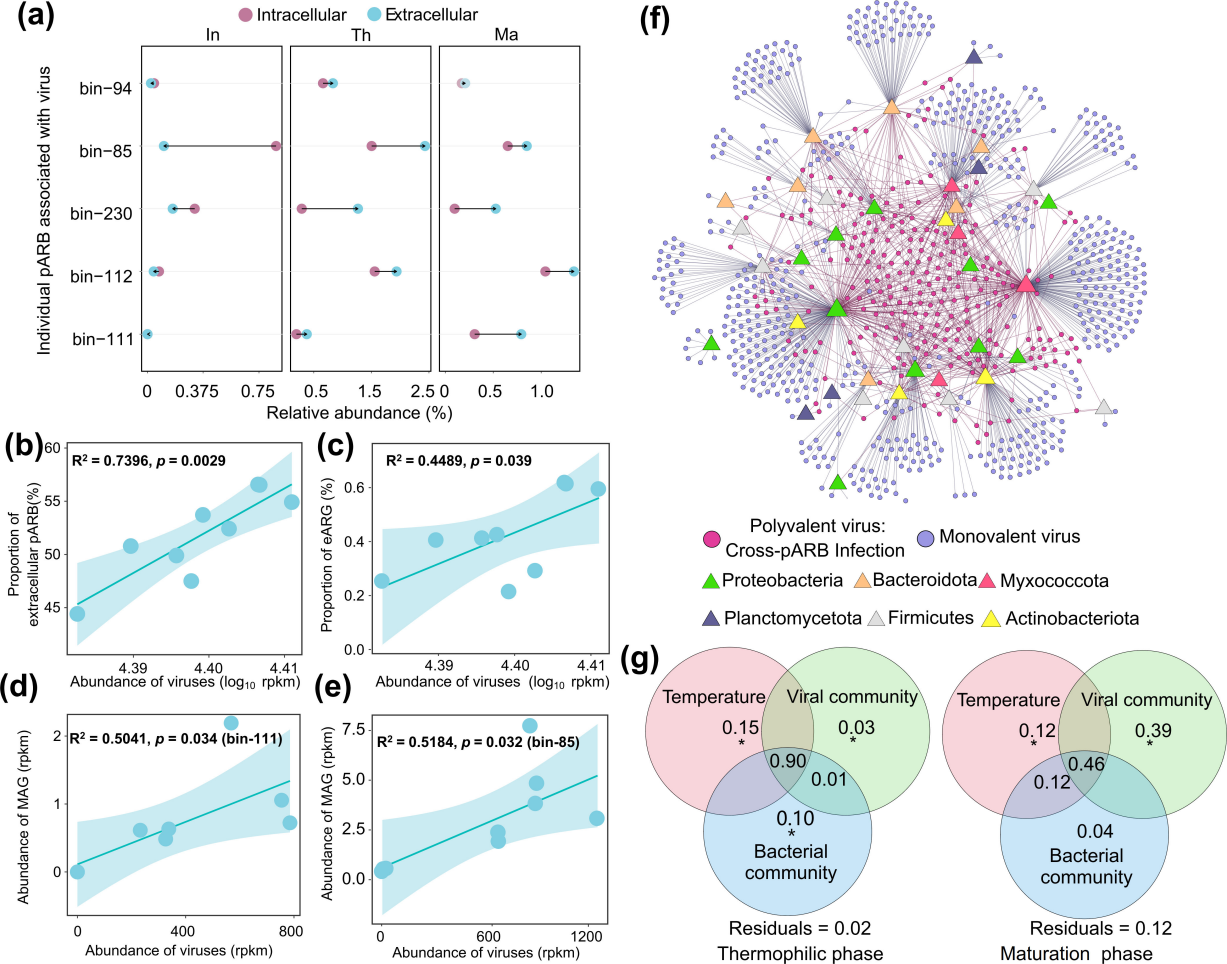
Lytic viruses mobilize eARGs during composting. (a) Associations between viruses and pARB host taxa (triangles colored by phyla) based on CRISPR spacers matching. The pink dots represent potential polyvalent viruses capable of infecting multiple pARB, while blue dots represent monovalent viruses associated with one host phylum. (b) Relationship between abundance of pARB-associated viruses and proportion of pARB in environmental DNA fraction averaged over all composting phases. (c) Relationship between abundance of pARB-associated viruses and proportion of eARGs across all composting samples. (d and e) Relationships between the environmental abundance of individual pARB (bin-111 and bin-85) and the associated viruses across all composting samples. (f) Virus-pARB interaction association based on CRISPR spacer matching. The pink dots represent polyvalent viruses capable of infecting multiple pARB, while the blue dots represent monovalent viruses. The other colored dots represent viral pARB hosts (at the phylum level). **(**g**)** Variance partitioning analysis showing the percentage of variation of eARG abundances explained by composting temperature and viral and bacterial community composition during thermophilic (left) and maturation (right) phases of composting (statistical signiﬁcance based on PERMANOVA test with 999 permutations; * *P* < 0.05 and **P < 0.01). The shaded area in panels b–e represents a 95% confidence interval around the fitted regression line.

To associate bacterial MAGs with viruses, a CRISPR spacer-based approach was used to predict potential viral hosts (see Materials and Methods). A total of 951 vOTUs could be associated with 62 MAGs as the potential hosts (Fig. 3d). Of these hosts, 87.1% (54 of 62) were predicted to infect the pARB (Table S6). The most prevalent vOTUs infecting pARB belonged to the family *Kyanoviridae* (31.1% vOTUs), *Herpesviridae* (17.9% vOTUs), and *Gclasvirinae* (13.1% vOTUs). Among these vOTUs, a remarkable 94.6% (900 of 951) were predicted to infect pARB belonging to the phyla Proteobacteria, Bacteroidota, Myxococcota, Bdellovibrionota, and Actinobacteriota. Furthermore, we observed significant correlations between viral and pARB community composition and richness, irrespective of whether ARGs were located in the intracellular (*R*^2^ = 0.8469, *P* < 0.05 for community composition; *R*^2^ = 0.9801, *P* < 0.05 for richness, Fig. 3e) or extracellular DNA fraction (*R*^2^ = 0.81, *P* < 0.05 for community composition; *R*^2^ = 0.9801, *P* < 0.05 for richness, Fig. 3e). To explore the interactions between viruses and pARB in more detail, a network of viruses infecting hosts was analyzed based on CRISPR spacer matches. Shared CRISPR spacer matches between phage and bacterial genomes provide high-confidence information on historical infection interactions between hosts and viruses (69, 70), and consequently, shared CRISPR spacers can be used to track virus transmission events (71). Of the 900 vOTUs, 35.8% (322 of 900) were identified to infect multi-pARB, indicating that these vOTUs were possible polyvalent viruses (Fig. 4a). For example, a lytic S6C1156 virus was predicted to infect 11 different pARB across four phyla (Acidobacteriota, Proteobacteria, Bacteroidota, and Myxococcota). One possible explanation for this is that because the rate of microbial community turnover is rapid during composting, being able to infect multiple host taxa could increase the chances of phage survival when the host availability fluctuates temporally (15). While viruses capable of between-phyla infections have been demonstrated in the soil (72), such viruses have not been documented in the compost ecosystems previously. We also observed that 5.9% vOTUs (53 of 900) were capable of infecting different pARB at different phases of composting. This may represent an adaptive strategy for the viruses (73), utilizing them to switch their preferred hosts along with the composting-induced bacterial community succession, enabling their long-term survival in changing environments (74). Together, these results suggest that viral and pARB community dynamics were coupled during composting, which is in line with our previous hyperthermophilic composting study (15).

To explore how viral lysis potentially impacted the eARG release, we focused on the dynamics of virus-host interactions using 900 vOTUs and their 54 pARB hosts. As expected, a highly significant positive correlation between the vOTUs and pARB proportion in environmental fraction was observed (*R*^2^ = 0.7396, *P* = 0.0029, Fig. 4b), suggesting that the increase in the proportion of eARGs could have been partly due to viral lysis of pARB, releasing eDNA into compost. In support of this, we found that most pARB (79.32%) associated with viruses exhibited a significant increase in the abundance of ARGs in eDNA fraction during thermophilic and maturation phases compared to the initial phase of composting (*F*_2,6_ = 113.5, *P* < 0.05). Specifically, the abundances of two dominant and multi-resistant pARB (bin-85 and bin-111) and their predicted viruses were positively correlated (bin-85, *R*^2^ = 0.5184, *P* < 0.05; bin-111, *R*^2^ = 0.5041, *P* < 0.05), suggesting that these viruses continuously lysed their ARG-carrying hosts (Fig. 4d and e). At the same time, the ARGs carried by these two dominant pARB (bin-85 and bin-111) became more abundant in the eDNA fraction during thermophilic and maturation phases (Fig. 4f). Similarly, we found that the eDNA fraction of ARGs carried by bin-42 (Proteobacteria with six ARGs) and bin-176 (Proteobacteria with nine ARGs) was significantly higher compared to cellular fraction during the thermophilic and maturation phases of composting (*P* < 0.05, *t* = 4.8, and df = 4). However, the above results are based on sequencing analysis only, and further experimental verification is hence required. Together, these results suggest that the viral lysis of pARB likely increased the eDNA fraction of ARGs during composting.

To disentangle the relative importance of viral lysis, composting temperature, and bacterial community composition for the eARG release during composting, a VPA was conducted (Fig. 4g). We found that viral lysis and composting temperature explained 90% and 46% of the total variance of eARG abundances, respectively. Specifically, the positive effect of viral lysis for the eARG abundances was relatively greater than the effect of temperature at the maturation phase, accounting for 39% (Mantel test, *P* < 0.05, *R* = 0.4107) of the total variance. In contrast, composting temperature (*P* = 0.0405, 15%) had a relatively greater effect on eARG abundances than viral lysis at the thermophilic phase (*P* = 0.0458, 3%). Given that mesophilic MAGs at the non-thermophilic phases of composting contained a relatively higher number of ARGs, viral lysis may be an important factor for the release of ARGs at the maturation phase of composting. This could partly explain the rebound and localization of ARGs in mesophilic pARB when the composting temperatures returned to ambient levels (75). Together, these results indicate that both viral lysis and composting temperature can drive eARG release during composting, with viral lysis seeming to be more pronounced after the thermophilic phase during the maturation phase of composting.

### eARGs released during the composting pose a potential transmission risk

To experimentally test whether eARGs released during composting can be taken up by naturally competent and potentially pathogenic bacteria, we measured the natural transformation efficiency of compost eDNA using *Vibrio vulnificus* as the recipient host. We found that compost eDNA could be successfully taken up by *V. vulnificus* to produce a tetracycline tolerant strain, and the highest transformation efficiency was observed with the eDNA isolated at the thermophilic phase of composting (Fig. S16). Consistent with previous studies (76, 77), our results further confirmed that eDNA derived from composting has the potential for transmission of ARGs via transformation. In contrast to eARG transformation measured in the activated sludge (78), we observed a 2.3-fold higher transformation efficiency at the thermophilic phase of composting, likely due to more efficient thermal killing of bacterial cells. More experiments with compost-isolated bacteria are, however, needed in the future to validate our results.

**Fig 5:**
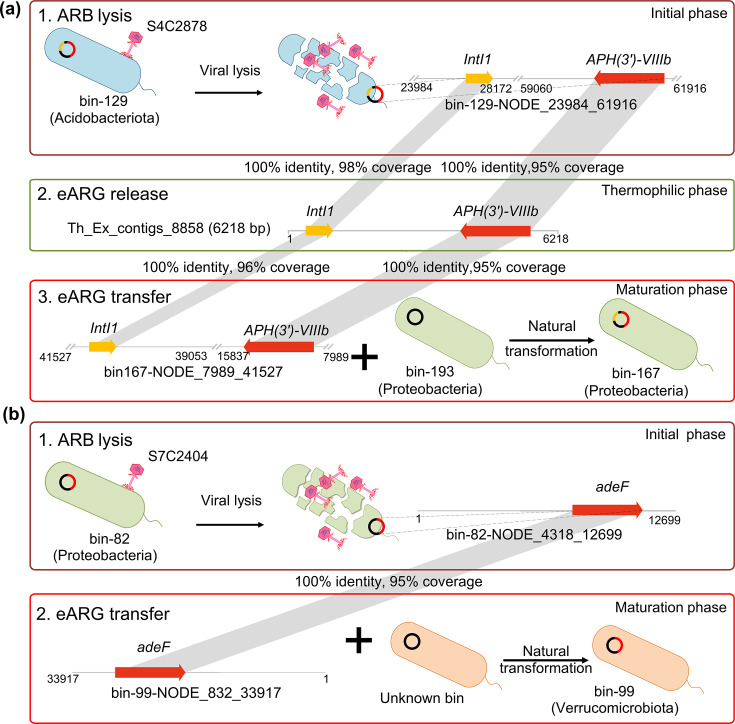
Summary of the fate of eARGs during composting based on the “viral lysis-absorb-uptake” model. The evidence for this conceptual model is based on two ARG-carrying contigs found across metagenome-assembled genomes in our data. (a) During the initial and thermophilic phases, an Actinobacteria MAG (bin-129, associated with one lytic virus) contains an intracellular ARG contig [*APH(3')-VIIIb*] and an integrase gene, which was also detected in the extracellular fraction of DNA. During the maturation phase, the same ARG contig is observed in the intracellular fraction of a Protobacterial MAG (bin-167). (b) Another ARG contig (*adeF*) present in the extracellular fraction of a Protobacterial MAG (bin-82, associated with one lytic virus) was observed in the intracellular fraction of a Verrucomicrobiota MAG (bin-99). Together, these results suggest that viral lysis likely helps cross-phylum transmission of antibiotic resistance genes during composting through natural transformation.

Based on metagenomics, we found that the richness of viable pARB significantly increased during the maturation phase of composting compared to the initial phase (Fig. S17), indicative of a clear change in the diversity of pARB communities during the composting. This result is supported by the isolation of culturable antibiotic-resistant bacterial strains from different phases of composting. At the species level, 54.1% of culturable antibiotic-resistant strains derived from the maturation phase were not present at the initial phase of composting (Fig. S18). At the phylum level, a tetracycline-resistant isolate *Microbacterium sorbitolivorans* belonging to Actinobacteria phylum was exclusively isolated at the maturation phase of composting. One of the possible reasons for the changes in the resistant bacterial community composition during different phases of composting could be the spread of ARGs. We tracked down example cases of ARGs that were found in MAGs in both intracellular and extracellular fractions at different phases of composting. We found one ARG [*APH(3')-VIIIb*] and MGE (*intI1*) within an Actinobacterial MAG (bin-129), which was present in both intracellular and extracellular DNA fractions at the initial phase of composting. As the composting temperature was close to ambient temperature at this time point, it is possible that viral lysis caused the release of this ARG into the eDNA pool. Interestingly, these two genes [*intI1* and *APH(3')-VIIIb*] were later observed in the intracellular fraction of another Protobacterial MAG (bin-167) during the maturation phase of composting, indicative of a potential horizontal gene transfer event. Moreover, we identified another MAG (bin-193) belonging to the same species as the Protobacterial MAG (bin-167) as a likely recipient strain for *APH(3')-VIIIb* gene as it was only detected in this MAG background only at the late phases but not during the initial phase of composting (Fig. 5). Finally, another ARG (*adeF*) was found to be carried in the extracellular fraction of a Protobacterial MAG (bin-82) at thermophilic phase and later in the intracellular fraction of Verrucomicrobal MAG (bin-99) at the maturation phase, indicative of potential between-phyla movement of ARGs. However, more evidence is needed to causally show the movement of ARGs between bacterial taxa, such as use of fluorescent antibiotic-resistant plasmids or isotope labeling to directly track horizontal gene transfer of eARGs. As these MAGs were also associated with several lytic viruses, it is possible that phages also drove the release and uptake of these eARGs between different bacterial genera (79). Interestingly, we found that several viruses were predicted to be linked with multiple hosts, indicative of polyvalence and broad host range. While viruses are generally thought to have narrow host ranges, recent studies have suggested that these broad host range viruses may have been overlooked due to insufficient cultivation and detection techniques (80–83). In addition, a recent study using plasmid *pX3_NDM-5* tagged with the *gfp* gene and cell sorting experiments demonstrated that plasmid-mediated transfer of ARGs is common across different bacterial phyla (84).

Based on these results, we propose a conceptual “viral lysis-absorb-uptake” model that could potentially explain eARG release and movement during composting through three following steps (Fig. 5) (85): (i) eARG release: eARGs are continually introduced into the composting matrix through cell death either due to viral infection, high temperature, or other factors causing bacterial mortality (14). (ii) eARG absorption: eARGs are bound to ubiquitous organic matter and thereby protected against degradation by DNases (86, 87). (iii) eARG uptake: eARGs are taken up and incorporated into the genomes of surviving bacterial taxa through natural transformation (e.g*.*, homologous recombination). Our results are in line with previous findings showing that as a result of microbial cell death, large fractions of eDNA are released into compost and soil environments, resulting in ARG enrichment (7, 75). Although several factors, such as environmental stress, could trigger bacterial cell death, viral lysis may be highly important as viruses are the most abundant entities on Earth. For example, our previous study found that mesophilic and thermophilic viruses are active during all phases of hyperthermophilic composting (15) and that viral and bacterial abundances are tightly coupled, indicative of strong top-down control of bacteria by phages. While phages have been used to treat multi-drug resistant infections in humans by specifically pathogenic host bacteria (88), the subsequent dissemination risk of ARGs via transformation and uptake of released eDNA has remained largely neglected. While viruses are known to drive the dissemination of ARGs via transduction, our study reveals a novel indirect method where viral lysis of bacteria releases ARG contigs in the environment as eDNA that can be up taken by surviving bacteria. As our results are specific to the composting system, it will be important to study if viruses will have similar roles in ARG transmission in other environmental and even clinical contexts. Crucially, in this system, both temperature and phage lysis were important for eDNA release, with temperature having a relatively more important effect at the thermophilic phase and viral lysis at non-thermal phases. Our result highlights the importance of eDNA release for the potential inter-phyla ARG dissemination in composting microbiomes, which should be considered when studying ARG movement and fate during composting. It is also crucial to consider how to mitigate the release and movement of eARGs and how eARGs could be removed during composting. To achieve this, new techniques such as high-temperature heat treatment could be implemented to degrade the eDNA and eARGs. Future research with additional experimental evidence is also required to causally demonstrate that viral lysis can facilitate the transmission of ARGs between bacteria during composting.

### Synopsis

Antibiotic resistance genes (ARGs) pose a substantial and growing threat to global health. This study reports previously unrecognized roles of thermal and phage lysis for ARG dissemination by releasing DNA into the environment.

## Data Availability

Raw read data generated from both amplicon and shotgun sequencing in this study have been deposited with the National Genomics Data Center under BioProject accession PRJCA018015.

## SUPPLEMENTAL MATERIAL

Supplemental figures aem.00695-24-s0001.docx Figures S1 to S18.

Supplemental tables aem.00695-24-s0002.xlsx Tables S1 to S6.

ASM does not own the copyrights to Supplemental Material that may be linked to, or accessed through, an article. The authors have granted ASM a non-exclusive, world-wide license to publish the Supplemental Material files. Please contact the corresponding author directly for reuse.
