# Supplementary material for: Viral and thermal lysis facilitates transmission of antibiotic resistance genes during composting
Source: Appl Environ Microbiol. 2024 Jul 30;90(8):e00695-24. doi: 10.1128/aem.00695-24 (PMC11337816; doi:10.1128/aem.00695-24)
Supplement: Supplemental figures — Figures S1 to S18. [file aem.00695-24-s0001.docx]

**Supplementary Information**

**Viral and thermal lysis facilitates transmission of antibiotic resistance genes during the composting**

Chaofan Ai^1#^, Peng Cui^2#^, Chen Liu^1^, Weijia Wu^1^, Yuan Xu^1^, Xiaolong Liang^3^, Qiu-e Yang^1^, Xiang Tang^1^, Shungui Zhou^1^, Hanpeng Liao^1*^, Ville-Petri Friman^4^

**^1^** Fujian Provincial Key Laboratory of Soil Environmental Health and Regulation, College of Resources and Environment, Fujian Agriculture and Forestry University, Fuzhou 350002, China.

**^2^** Guangdong Provincial Engineering and Technology Research Center for Agricultural Land Pollution Prevention and Control, Zhongkai University of Agriculture and Engineering, Guangzhou 510225, China.

**^3^** Key Laboratory of Pollution Ecology and Environmental Engineering, Institute of Applied Ecology, Chinese Academy of Sciences, Shenyang, Liaoning Province 110016, China

**^4^** Department of Microbiology, University of Helsinki, Helsinki 00014, Finland.

**^#^** Chaofan Ai and Peng Cui contributed equally to this work.

**^*^Corresponding authors**

Correspondence to Hanpeng Liao (liaohap@fafu.edu.cn)

**This supplementary information file contains 18 figures and 6 tables titles.**


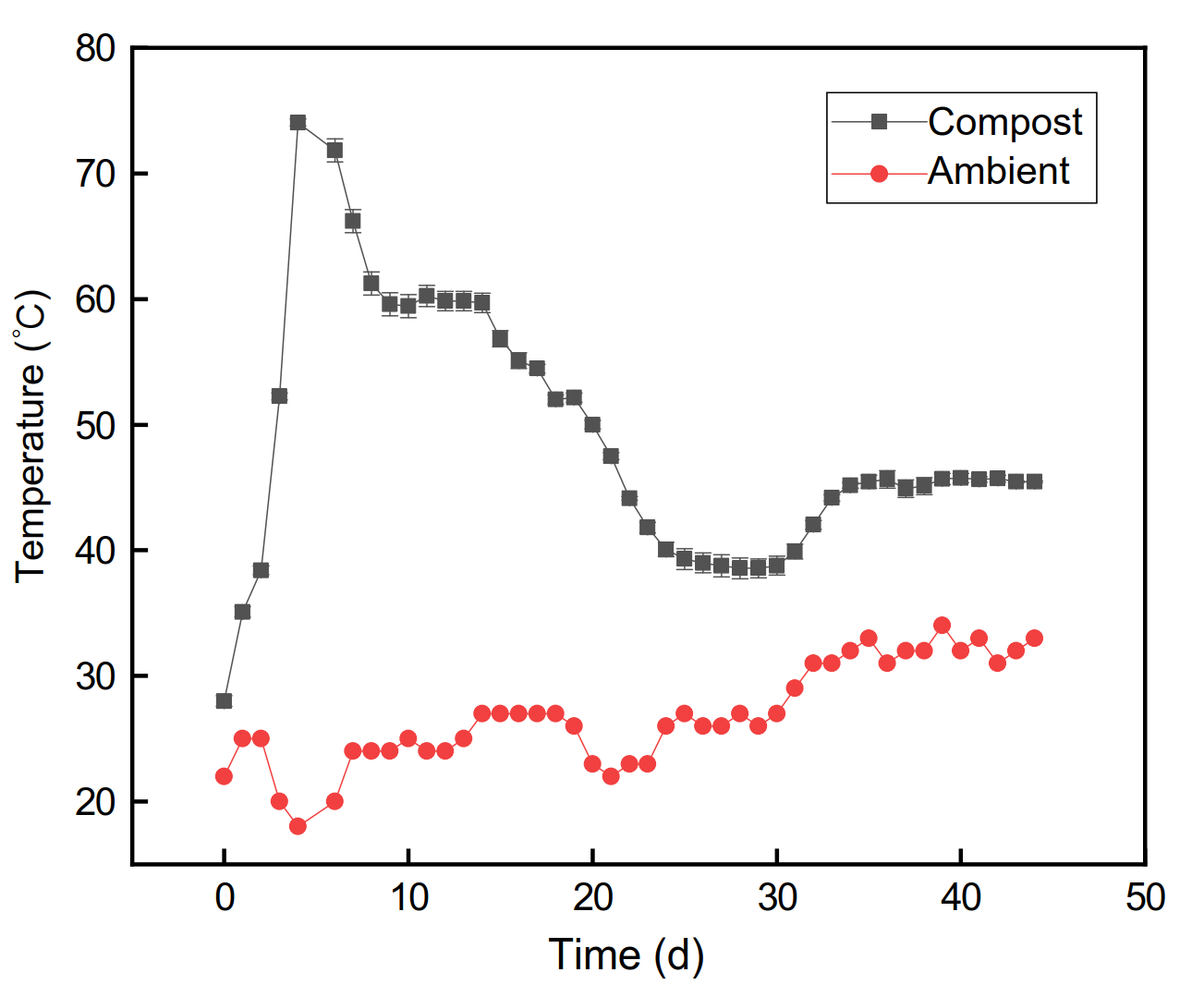


**Figure S1. Temperature variation during thermophilic cow manure composting**. The black and red curves represent composting and ambient temperatures, respectively (N=3).


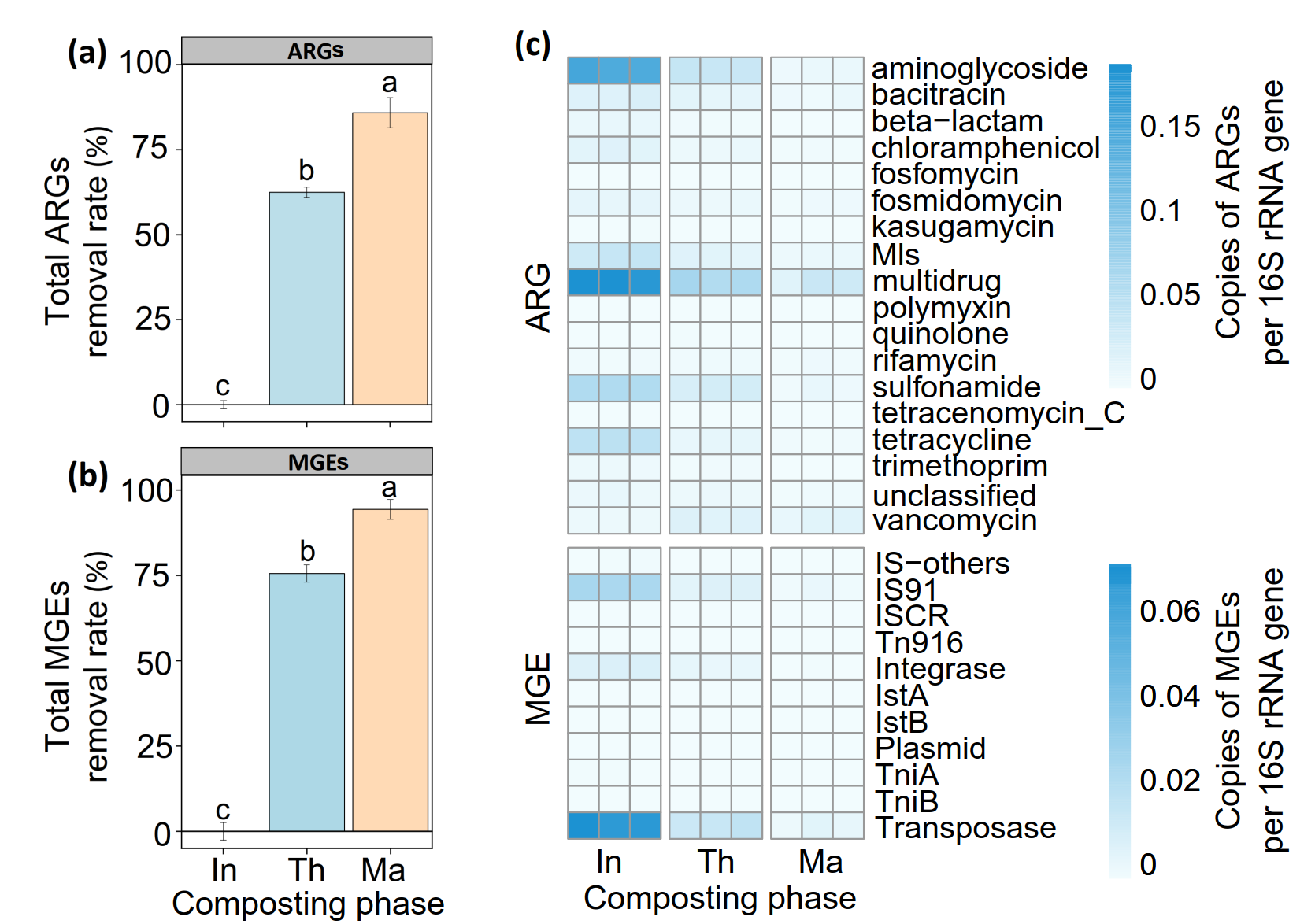


**Figure S2.** **Dynamics of total ARG and MGE abundances during composting.** **(a-b)** Removal rate of total ARGs and MGEs during different phases of composting. **(c)** Heatmaps representing the mean abundances of individual ARG and MGE types during different phases of composting. In a, b, and c, different letters represent significant differences between composting phases (*p* < 0.05). The ‘In’, ‘Th’ and ‘Ma’ represent the initial, thermophilic, and maturation phases, respectively (N=3).


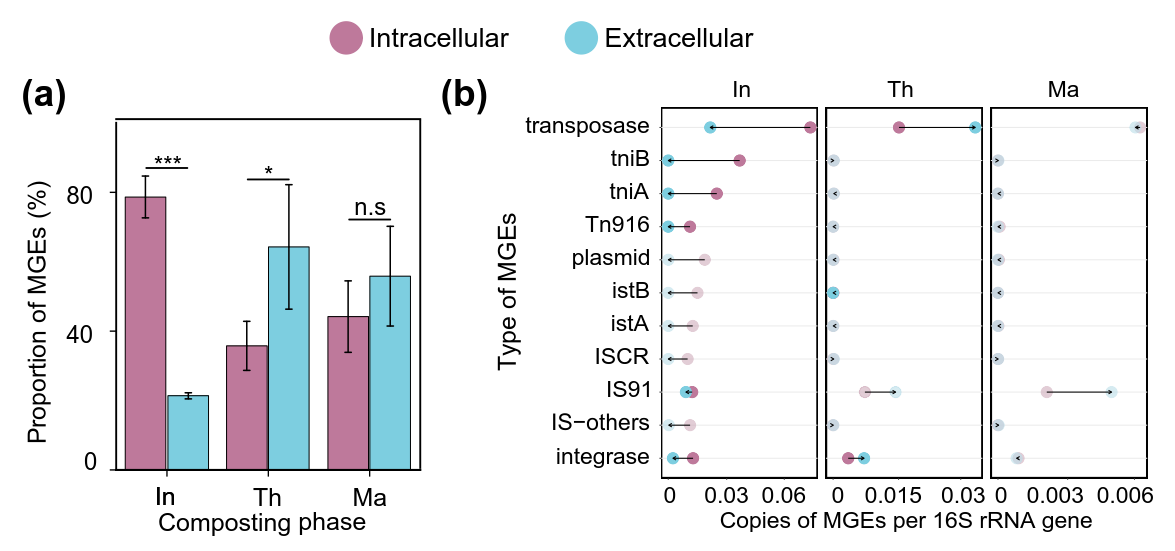


**Figure S3. Variation in the intracellular and extracellular MGEs during composting.** **(a)**: Changes in the proportion of intracellular and extracellular MGEs at different phases of composting (‘In’, ‘Th’ and ‘Ma’ represent the initial, thermophilic and maturation phases of composting, respectively). **(b)**: Dumbbell plots illustrating the abundance differences of intracellular and extracellular MGEs during composting. Dumbbells with dark colors indicate significant differences between groups (*p* < 0.05), while lighter colors indicate non-significant differences (*p* > 0.05). All datasets are based on three biological replicates and significance levels are indicated by* (*p* < 0.05), ** (*p* < 0.01), and *** (*p* < 0.001), while ‘n.s’ denotes non-significant difference (*p* > 0.05).


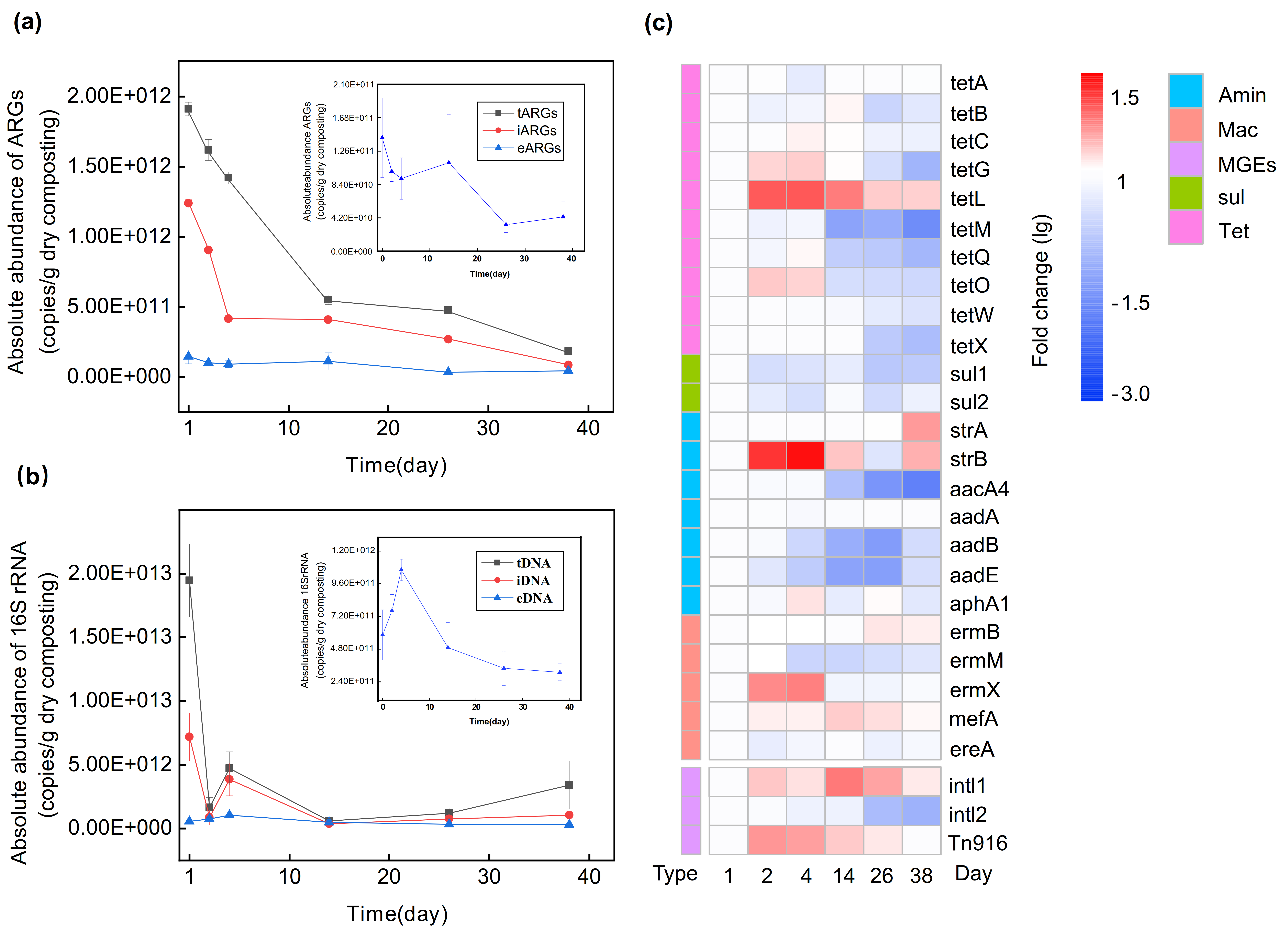


**Figure S4. Changes in absolute abundance of total ARG (a), 16S rRNA gene (b), and individual ARG (c) during composting. Heatmap (c) showing the fold changes of individual eARG and eMGE abundances relative to the initial phase of composting (day 1).** The data is based on qPCR. In panel (c), the value shown were log10-transformed.


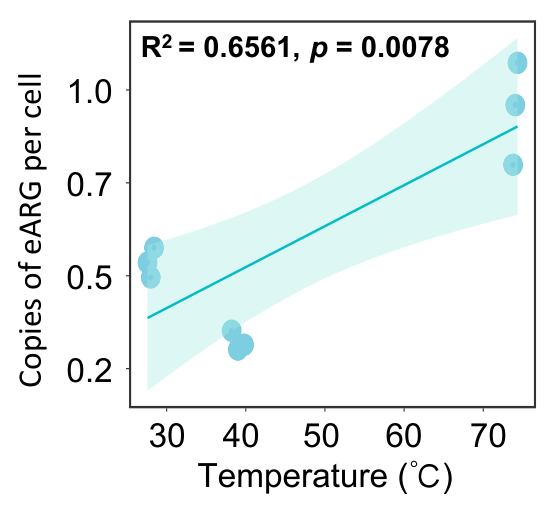


**Figure S5. The relationships between** **eARG abundances with composting temperature.** The shaded area in panel represents a 95% confidence interval around the fitted regression line.


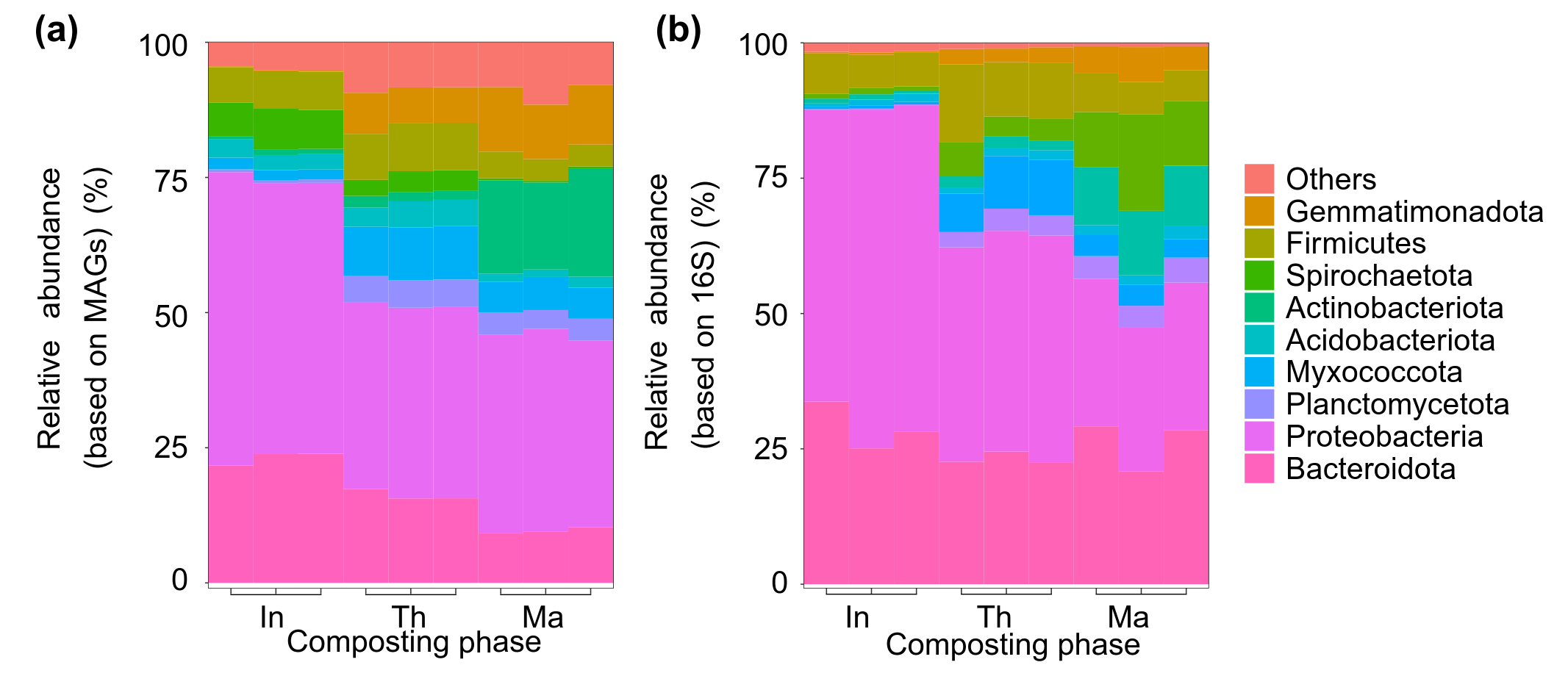


**Figure S6. Variation in bacterial community composition (phyla level) based on metagenomic MAGs (a) and 16S high-throughput sequencing data (b) during composting (N=3).**


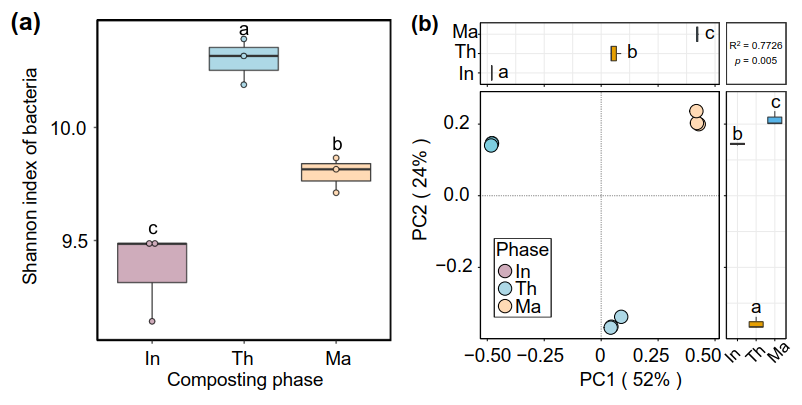


**Figure S7. Dynamics of bacterial alpha diversity (Shannon index) and community composition during composting.** **(a)** Shannon index of bacterial communities during composting. **(b)** Principal coordinate analysis (PCoA) based on weighted Unifrac distances for bacterial community composition during composting. The differences in community composition were compared using nonparametric multivariate ANOVA (PERMANOVA, Adonis function, 999 permutations). Different letters (a, b, and c) represent significant differences between groups (*p* < 0.05). The ‘In’, ‘Th’ and ‘Ma’ represent the initial, thermophilic, and maturation phases, respectively (N=3).


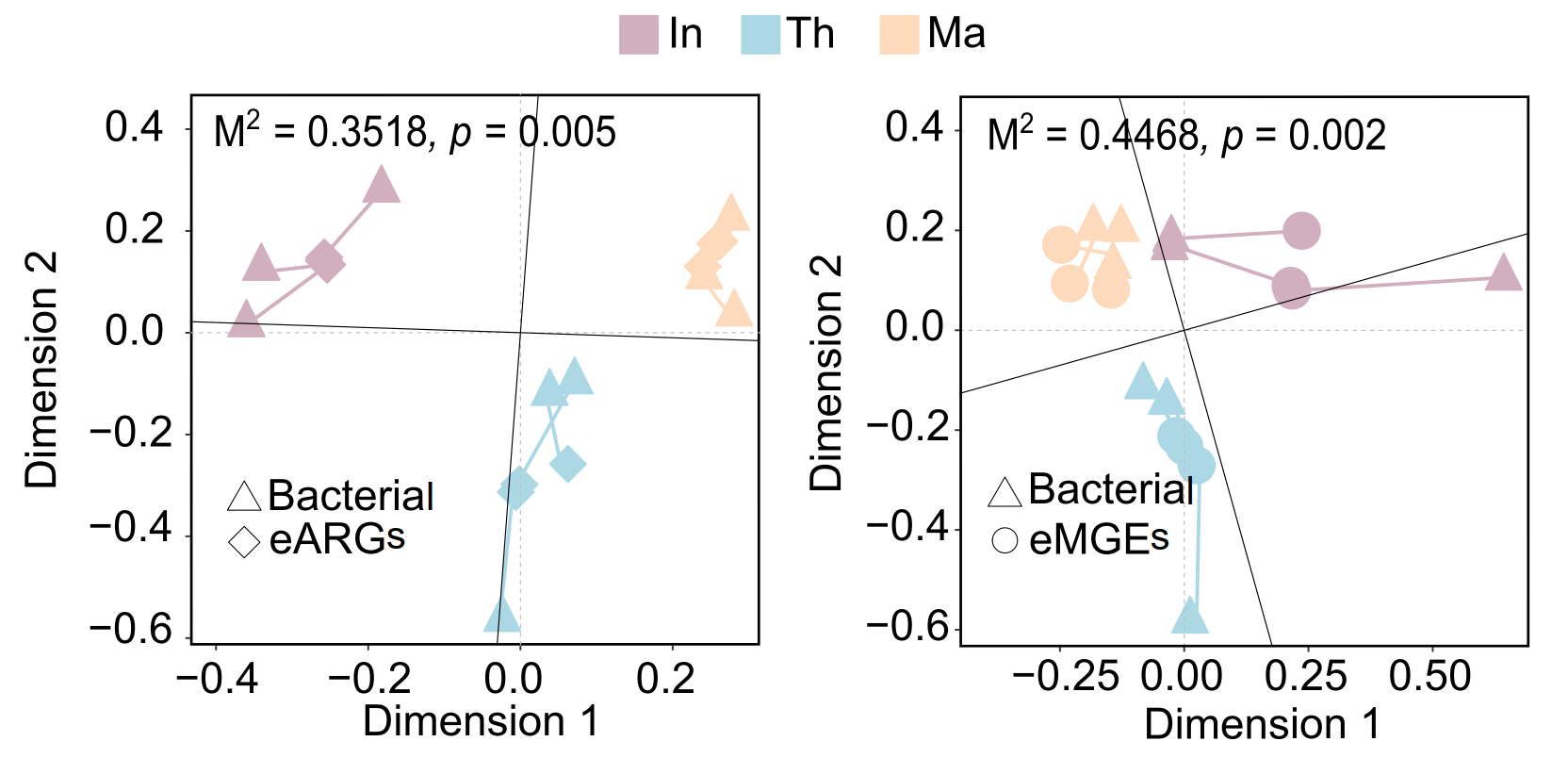


**Figure S8. The relationships between bacterial community composition with eARGs and eMGEs.** Procrustes analysis showing signiﬁcant correlations between changes in eARGs (left) and eMGEs (right) with bacterial community composition (16S rRNA sequencing) based on the Bray−Curtis dissimilarity metrics (N = 3).


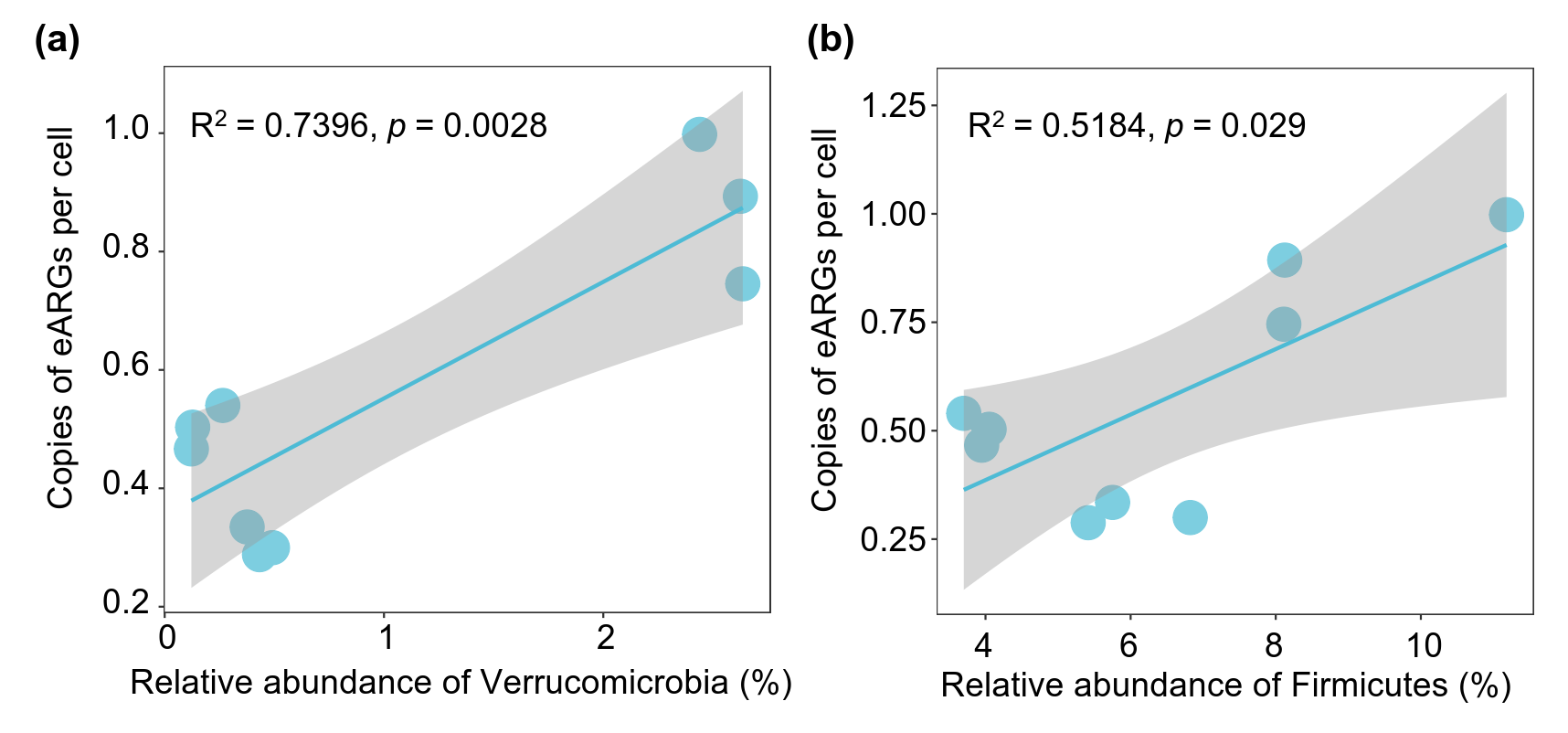


**Figure S9. Positive relationships between extracellular Verrucomicrobia and Firmicutes and eARG abundances. (a)** Correlation between extracellular abundances of Verrucomicrobia and eARGs. **(b)** Correlation between extracellular abundances of Firmicutes and eARGs. The shaded area in panels represents a 95% confidence interval around the fitted regression line.


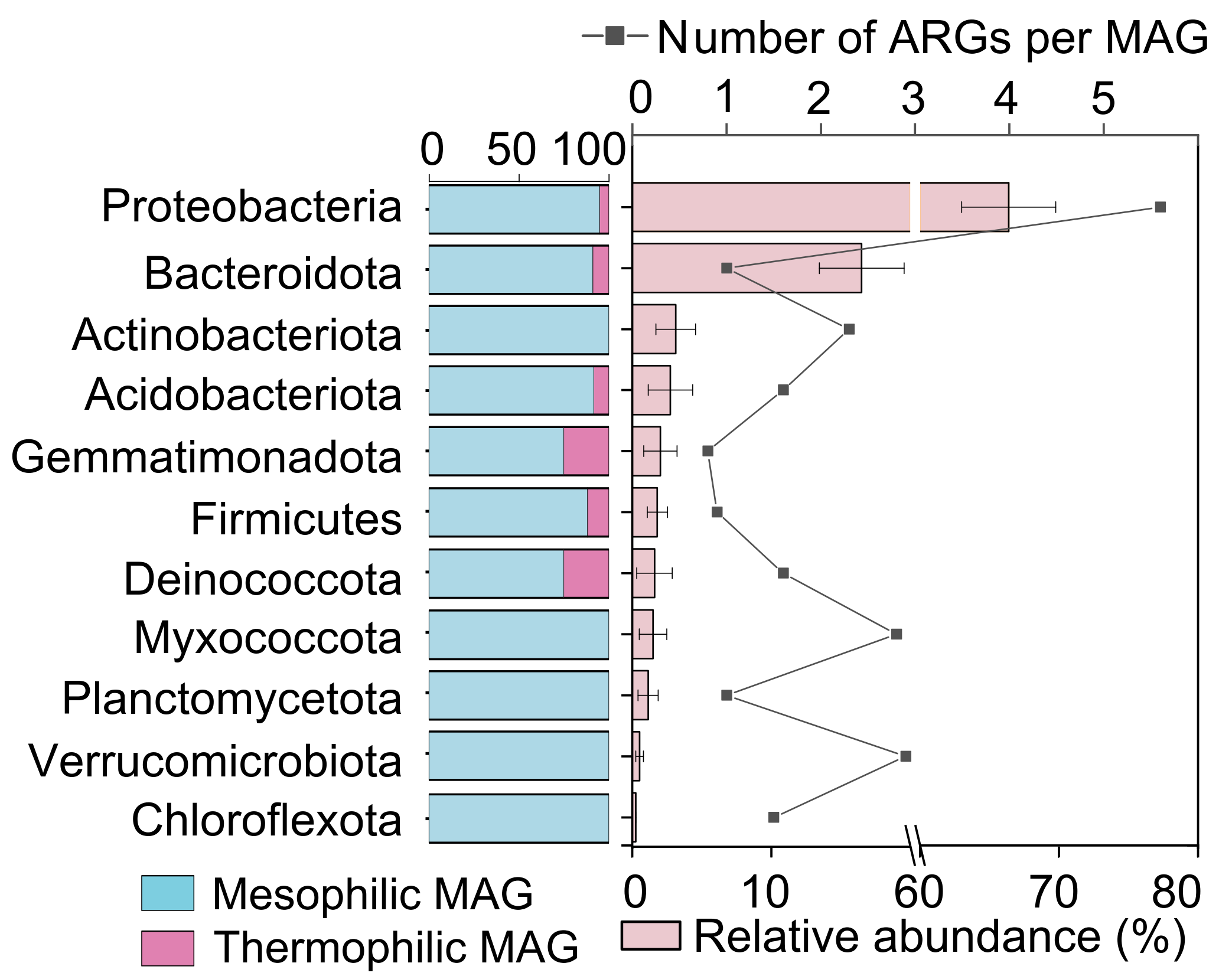


**Figure S10. The relative abundances and ARG profiles of MAGs at the phylum level.** Colors on the left panel shows the proportion of mesophilic and thermophilic MAGs.


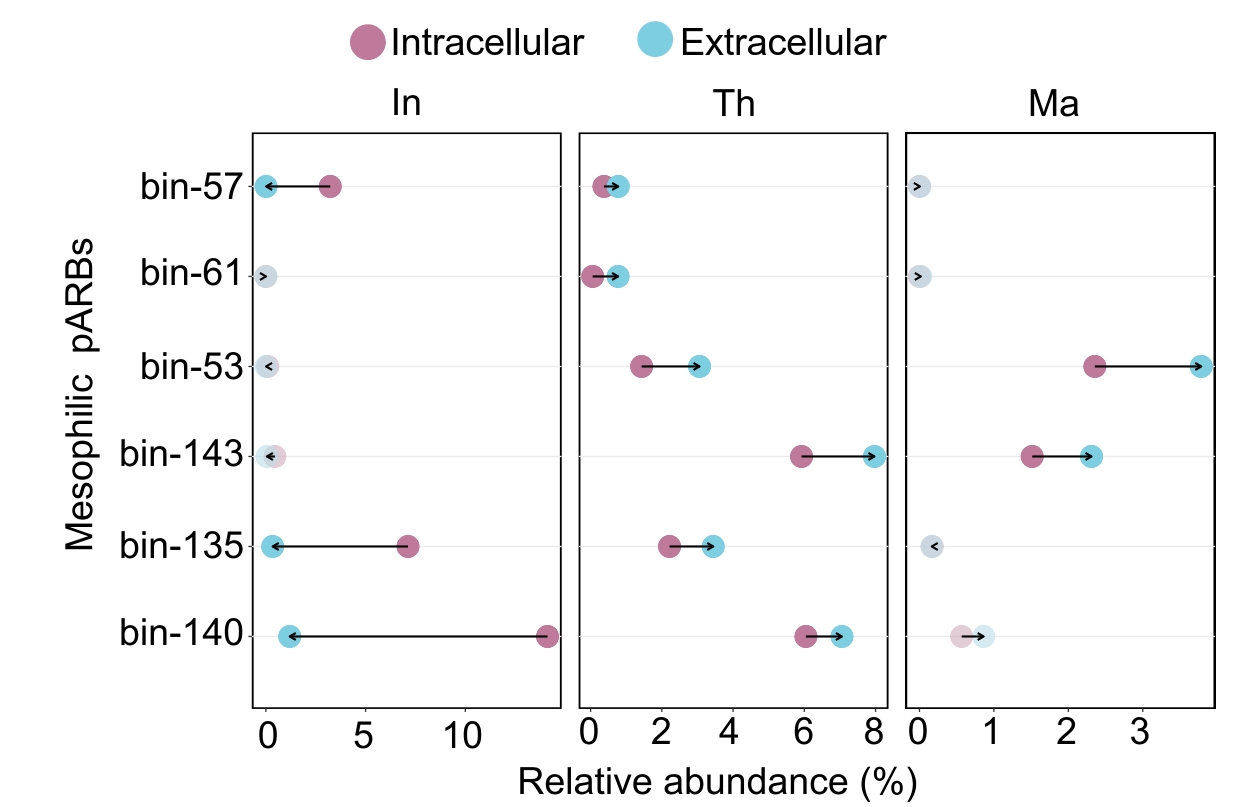


**Figure S11. Variation in cellular DNA and environmentally released DNA of mesophilic pARBs.** Dumbbell plots illustrate the abundance differences of cellular DNA and DNA released in the environment due to cell lysis during composting. Dumbbells with dark colors indicate significant differences between different fractions (*p* < 0.05), while lighter colors indicate non-significant differences (*p* > 0.05). The ‘In’, ‘Th’ and ‘Ma’ represent the initial, thermophilic, and maturation phases, respectively (N=3).


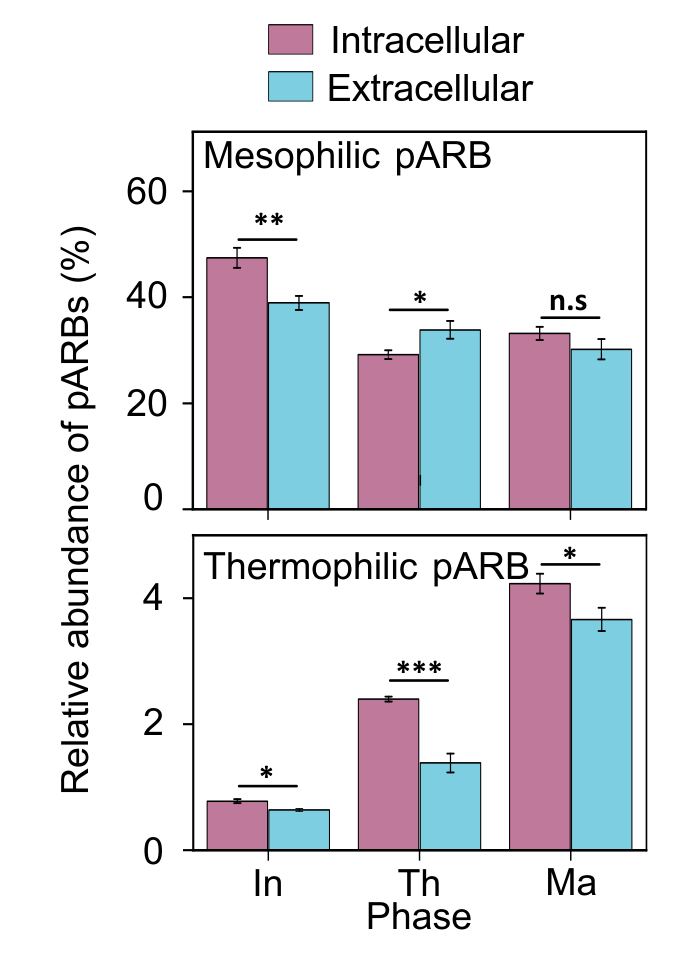


**Figure S12. Variation in the relative abundance of thermophilic (predicted OGT > 50 ^o^C) and mesophilic (predicted OGT < 50 ^o^C) pARBs observed as cellular DNA and environmentally released lysed DNA during composting.** Significance levels are indicated by * (*p <* 0.05), ** (*p <* 0.01), and *** (*p <* 0.001), ‘n.s’ denotes for nonsignificant difference (*p* > 0.05). The ‘In’, ‘Th’ and ‘Ma’ represent the initial, thermophilic, and maturation phases, respectively (N=3).


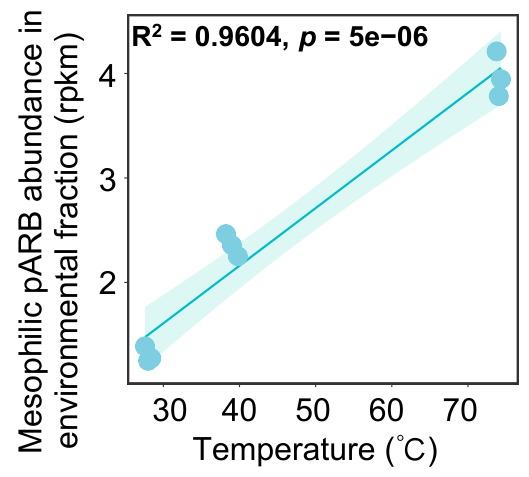


**Figure S13. Correlation between the abundance of pARBs in environmental DNA fraction (the abundance was determined after standardizing the concentration of eDNA) and temperature.** The shaded area in panel represents a 95% confidence interval around the fitted regression line.


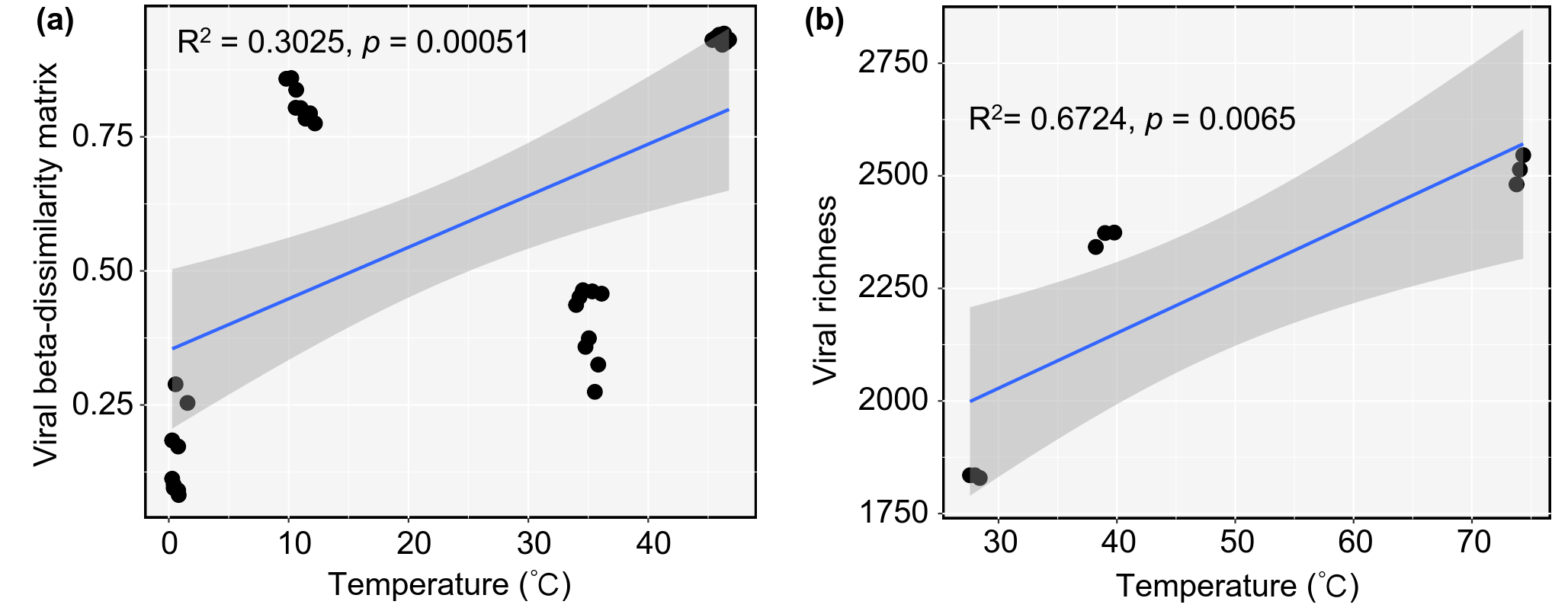


**Figure S14. Positive relationships between viral community beta and alpha diversities and composting temperature.** Panel (**a**) community beta-dissimilarity indexes and panel (b**)** richness indexes (N=3). The shaded areas in both panels represent 95% confidence intervals around the fitted regression lines.


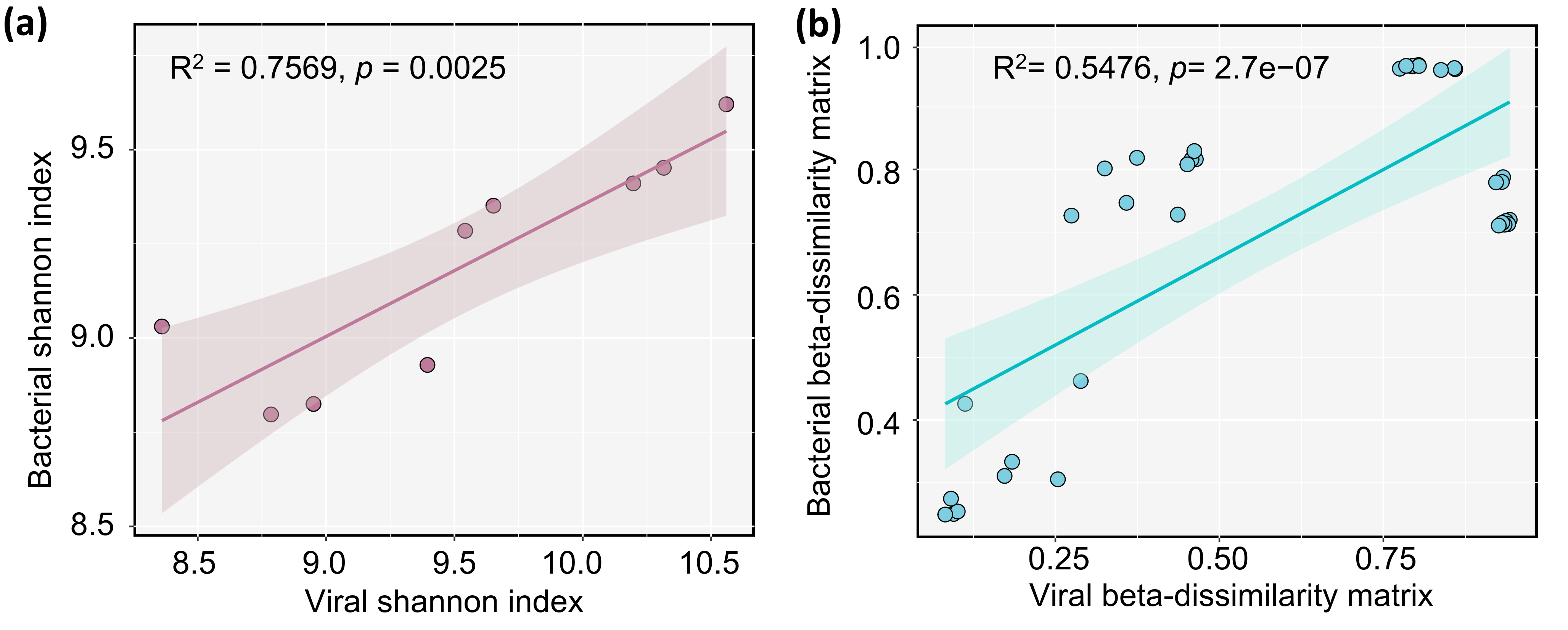


**Figure S15.** **Positive relationships between bacterial (16S rRNA amplicons sequencing) and viral taxa (vOTUs) alpha and beta diversities averaged over all composting samples.** Panel (**a**) compares alpha diversities in terms of Shannon indexes and panel (b**)** community beta-dissimilarity indexes (Bray-Curtis, N=3). The shaded areas in both panels represent 95% confidence intervals around the fitted regression lines.


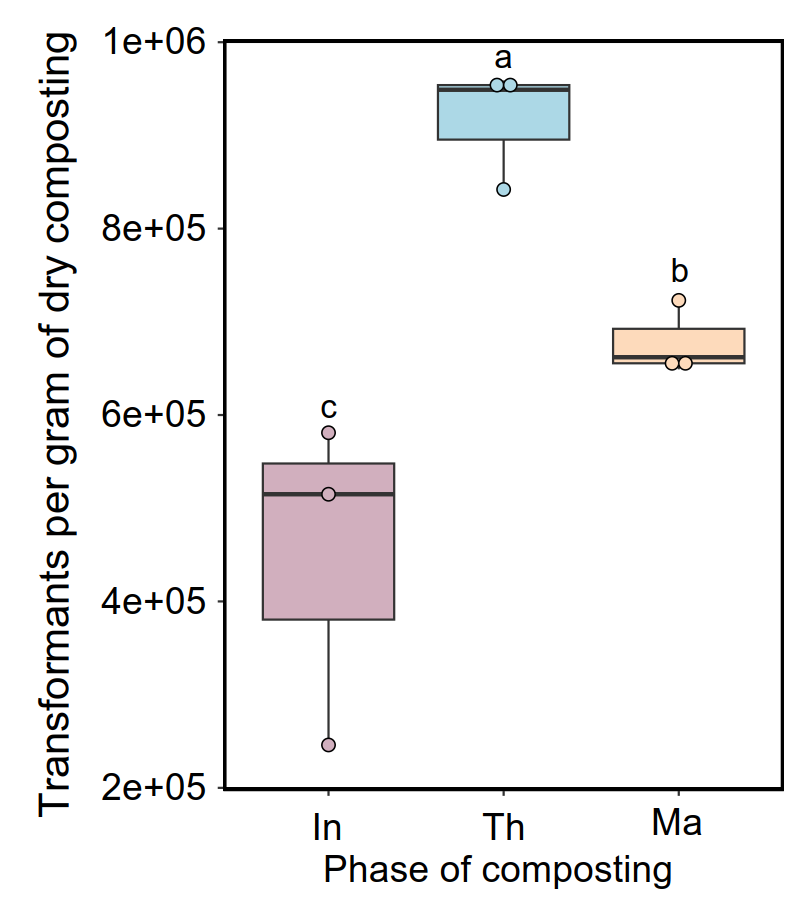


**Figure S16. Transformation efficiency of eARGs derived from different phases of composting using** ***Vibrio vulnificus* as the recipient host.** Different letters (a, b, and c) represent significant differences between groups (*p* < 0.05). The ‘In’, ‘Th’ and ‘Ma’ represent the initial, thermophilic, and maturation phases, respectively.


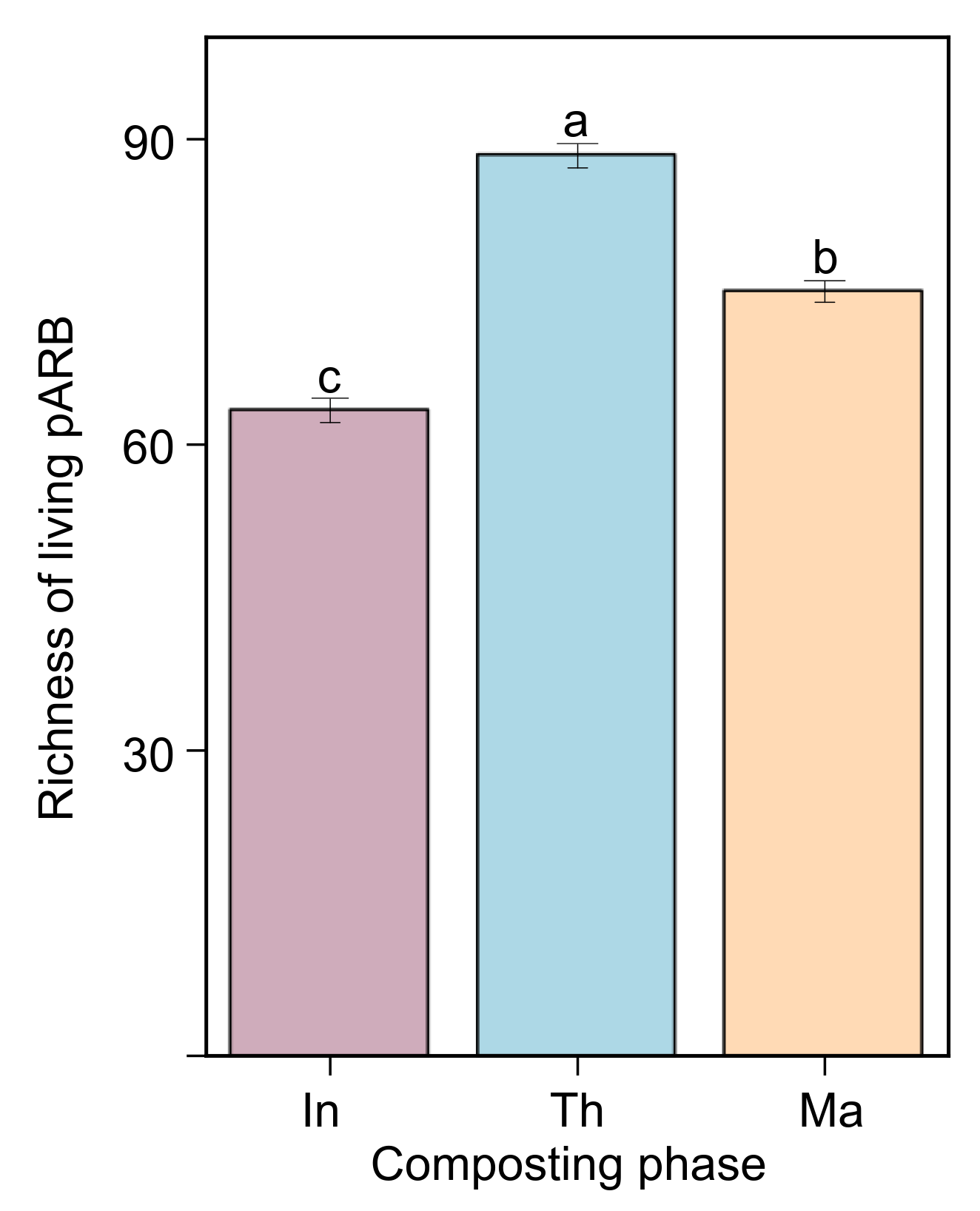


**Figure S17. Changes in the richness of living ARBs during composting.** Variation in the richness of non-lysed pARBs during composting. Different lowercase letters represent significant differences between groups (*p <* 0.05). The ‘In’, ‘Th’ and ‘Ma’ represent the initial, thermophilic, and maturation phases, respectively.


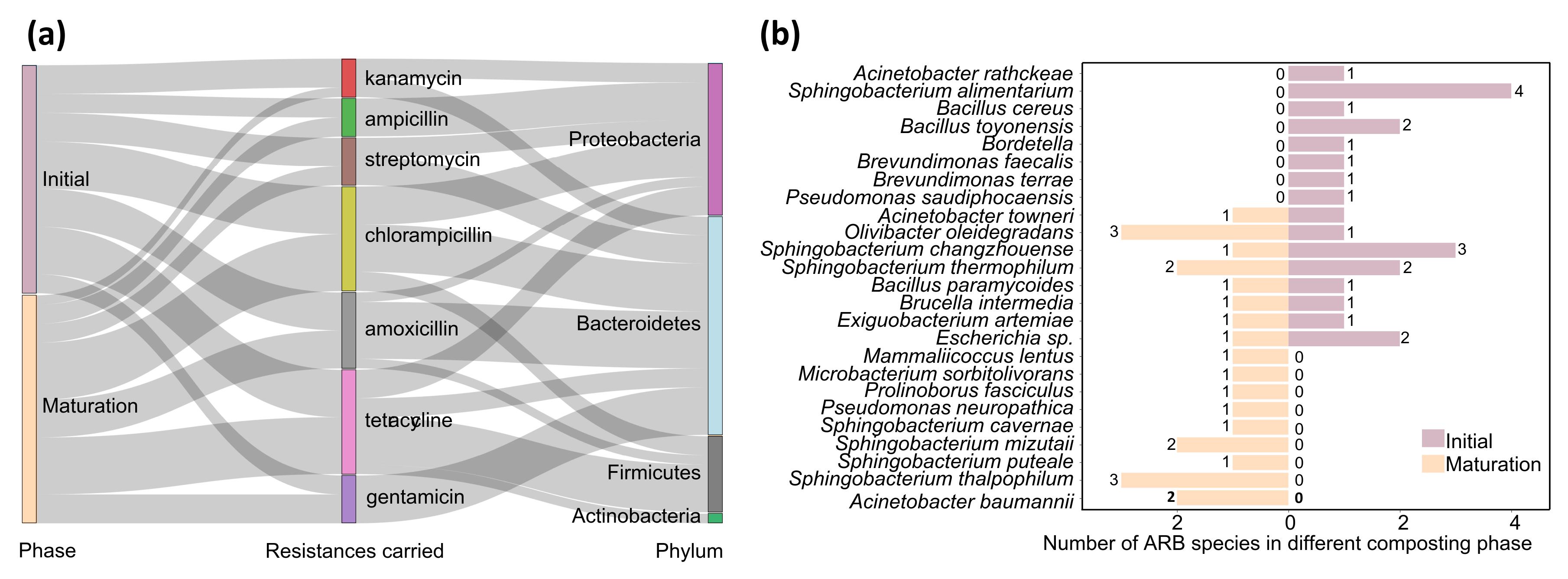
 **Figure S18. The antibiotic resistances and taxonomy of culturable antibiotic resistance bacterial isolated at the initial and maturation phases of composting.** (a) The first column on the left illustrates the composting phases where ARBs were isolated, the middle column shows the associated antibiotic resistance types, and the right column illustrates the taxonomic classification of ARBs at the phyla level. (b) The number of isolated culturable ARBs (at species level) at the initial and maturation phases of composting.

**Supplementary Tables**

**Supplementary Table S1 |** PCR primers used for targeting different antibiotic resistance genes (ARGs), mobile genetic elements (MGEs) and bacterial 16s rRNA gene in qPCR analyses [1-16].

**Supplementary Table S2 |** Detailed information on culturable antibiotic resistant bacteria at the initial and maturation phases of composting.

**Supplementary Table S3 |** Detailed information on MAGs identified in all composting samples.

**Supplementary Table S4 |** Basic information on identified ARGs present in all MAGs.

**Supplementary Table S5 |** Detailed information on predicted viral genomes identified in all composting samples.

**Supplementary Table S6 |** Virus-host linkages predicted by CRISPR spacer matches.
